# Self-perceived competencies of Master of Public Health graduates and recommendations for programs in South Africa

**DOI:** 10.1371/journal.pone.0360789

**Published:** 2026-10-16

**Authors:** Virginia Zweigenthal, Nicola Christofides, Thembelihle Dlungwane, Sogo France Matlala, Mathildah Mpata Mokgatle, Abraham Opare, Sean Mark Patrick, Nikki Schaay, Maylene Shung-King, Takalani Tshitangano, Laetitia Rispel

**Affiliations:** 1 School of Public Health, University of Cape Town, Cape Town, South Africa; 2 School of Public Health, University of the Witwatersrand, Johannesburg, South Africa; 3 Discipline of Public Health Medicine, University of KwaZulu-Natal, Durban, South Africa; 4 Department of Public Health, Sefako Makgatho Health Sciences University, Pretoria, South Africa; 5 School of Health Systems and Public Health, University of Pretoria, Pretoria, South Africa; 6 School of Public Health, University of the Western Cape, Cape Town, South Africa; 7 Department of Public Health, University of Limpopo, Polokwane, South Africa; 8 South African Research Chairs Initiative, South Africa; SPHMMC: St Paul’s Hospital Millennium Medical College, ETHIOPIA

## Abstract

The Master of Public Health (MPH) degree is a key qualification for public health practice in low- and middle-income countries. Graduates entering practice should possess a range of competencies to address complex health issues in managerial, technical or research positions. As competencies of graduates exiting South African MPH programs are unknown, a consortium of academics from South African universities conducted an on-line survey of alumni exiting between 2012−2016, to determine their self-reported public health competencies and their recommendations for improving academic programs. Competencies were explored in 11 domains, through three lenses – their reported usefulness at work; perceptions whether they were obtained through MPH programs; and graduates’ confidence in their application. Over 80% of this cohort of 221 graduates, mostly in their mid-30’s, largely from South Africa and sub-Saharan Africa, needed the competencies in all 11 domains at work, which were felt to be largely obtained from the MPH, and most were confident using them. However, one fifth were not confident in crisis management and 13% were not confident in health promotion. On average 30% reported they did not obtain either crisis management or leadership skills from the MPH. Respondents recommended changes to course content and organization included more experiential learning, mentorship and faculty advocacy for graduate employment. In the absence of country-wide governance for public health education, the study is a first step towards developing core-competencies for South African programs. Findings can be factored into MPH program reviews with attention given to enhancing leadership, crisis management and monitoring and evaluation training into existing courses. Additionally, more experiential learning could fill identified gaps in problem-solving and project management skills. Producing a ‘fit for purpose’ public health workforce requires alignment of education objectives with health workforce systems in country contexts to ensure a seamless transition between learning and working.

## Introduction

A skilled health workforce is required to embed public health functions in countries’ health systems, facilitating initiatives to keep populations healthy [1] and improving their health status [2]. This workforce also contributes to clinical health service delivery, necessary for realizing the health related Sustainable Development Goals (SDGs) and universal health coverage [3]. As the World Health Organization (WHO) notes, core public health personnel collaborate with health care workers and professionals allied to human health (such as engineers), to deliver essential public health functions (EPHFs).

Despite this, core public health personnel has been neglected in many countries’ health systems [4] in part due to the focus on clinical service delivery [5], the diversity of public health [6] and its invisiblity [7]. Ironically, public health becomes visible during disasters or health crises that are preventable or unexpected such as the COVID-19 pandemic [8]. The pandemic highlighted public health, its workforce, and the need for resilient health systems [9,10]. In Africa, the COVID-19 pandemic exposed how gaps in public health expertise resulted in inadequacies in surveillance, outbreak management and communication [11]. Consequently, the WHO embarked on a public health project that defines EPHFs, competencies needed for these functions and roadmap that serves to measure countrywide capacity [3].

In low- and middle-income countries (LMICs), public health training has focused on Master of Public Health (MPH) as the main qualification for public health practice, reflected in an increasing number of institutions that offer MPHs and a growing numbers of graduates [12].

Competency-based education is an approach that aims to ensure that graduates exit educational programs with measurable outcomes in knowledge, behavior, skills as well as attitudes [13]. The focus is on graduates’ ability to mobilize skills and qualities to successfully perform in particular contexts [14].

The MPH degree, introduced by several universities in South Africa over the last three decades [15], has no nationally agreed upon set of competencies. Universities determine the content of course work, which is accredited and quality assured by mandated educational authorities [16]. In addition to teaching traditional public health disciplines, there has been a proliferation of MPH tracks in specialist areas such as rural health, maternal and child health, environmental health and social behavioral communication, in response to the growth and breadth of public health and the diverse research and expertise of course coordinators or educators.

However, there are knowledge gaps on specific competencies attained across the various MPH programs in South African and whether graduates share a common core skill set upon completion. While a previous study of University of KwaZulu-Natal (UKZN) MPH graduates reported that they had acquired skills in report writing, critical analysis, research, leadership, and management [17], there is limited educational research that examined the competencies of alumni of South Africa’s MPH programs. Consequently, a consortium of public health academics from eight South African universities that graduated MPH students between 2012 and 2016 conceptualized an overarching mixed methods study to describe the socio-demographic characteristics of graduates, their career paths, competencies, leadership skills and impacts at work and in society. Their demographic characteristics, career trajectories, perceived competencies, leadership skills and perceived impacts as well as recommendations about MPH graduates’ roles are reported elsewhere [18,19]. This paper reports on graduates’ perceptions about selected public health competencies through three lenses: their usefulness at work, the extent to which these were obtained from the MPH; their confidence in applying them; and their recommendations to improve MPH programs In South Africa.

## Methods

This was a descriptive, cross-sectional, collaborative study among all eight South African university public health departments that graduated MPH students between 2012 and 2016. Universities that participated in the collaborative study were Sefako Makgatho Health Sciences University (SMU), the University of Cape Town (UCT), UKZN, University of Limpopo (UL), University of Pretoria (UP), University of Venda (Univen), University of the Western Cape (UWC), and the University of the Witwatersrand (Wits). During the study focus period, SMU and UL were combined as one university that taught the same curriculum at two campuses in two different towns. Hence, we merged the results from these alumni. Universities with newer or inactive programs were excluded.

Each university developed a list of eligible alumni and their contact details. Recruitment into the study was delayed by the COVID-19 pandemic, and the on-line survey was undertaken from 2022 until 2023. Consequently, alumni had between six and eleven years post-graduation experience which allowed for substantial work experience and reflection on their competencies. The survey consisted of closed-end statements using Likert scales and open-ended questions, allowing for free comment.

### Development of the competency tool

Prior to conceptualization of the study, our consortium members held informal discussions on MPH programs at the 2015 and 2016 annual conferences of the Public Health Association of South Africa. In 2018, when the study was conceptualized, we adapted Zwanikken *et al*.’s 2014 approach [20] which had included one South African university to develop our competency framework. Their validated instrument for LMIC settings is based on the literature, modified Delphi panels among experts and alumni, which yielded a high degree of consensus, suggesting that their set of competencies has content validity and could be used to design and evaluate MPH programs.

Although Zwanikken *et al.* acknowledged the US Linkages framework, they gave greater prominence to those public health competencies that incorporated the complexity and context of LMICs. Additionally, Zwanikken *et al.,* combined a review of the stated competencies of their six institutions, consensus building and validation of their tool focusing on self-perceived competence to arrive at seven competency categories [21]. Our tool built on Zwanikken *et al*.’s tool, and added questions derived from previous questionnaires used in MPH alumni studies in South Africa [17,22], and the experiential knowledge and curricula of investigators from the eight participating institutions. We also considered traditional public health curricula which have been organized around five core disciplines: biostatistics, epidemiology, environmental health, health policy and management, and social and behavioral sciences [23].

The tool was piloted among MPH graduates participating in our qualitative study and with MPH alumni from all eight institutions who were not part of the cohort of interest to determine face validity. Following the pilot, competencies in project management, financial literacy and managing crises were added to the tool. The final self-administered questionnaire (SAQ) was housed in and administered through REDCap (Research Electronic Data Capture v 13.1.37), a secure web application for managing online surveys and databases [24].

The SAQ included items about graduates’ demographics, educational backgrounds, career paths and recommendations for graduate roles in the light of COVID-19. Their demographics, educational backgrounds, career paths, leadership skills and perceived impact at work and in society are reported elsewhere [18,19].

The final SAQ examined 58 self-perceived competencies in 11 domains: 1) Public health sciences, 2) Health promotion, 3) Monitoring and evaluation (M&E), 4) Health policy, 5) Communication, 6) Context sensitivity, 7) Community and intersectoral skills, 8) Planning and management, 9) Crisis management, 10) Academic, and 11) Leadership. M&E became a separate domain as analysis of data from routine service delivery is the foundation of core public health work in the public sector in the South African setting. The ability to manage crises emerged as an important domain following the COVID-19 pandemic. As detailed in Table 1, each domain had between two and seven statements and Zwanniken *et al*.’s categories are indicated.

**Table 1:** The eleven competency domains with their specific activities.

Domain	Items tested
Public Health Sciences*	1. Apply biostatistics and epidemiology to public health policies and programs 2. Apply environmental health to public health policies and programs 3. Apply health service management skills to public health policies and programs 4. Apply public health approach to local context health system 5. Analysis of health status, health problems, risk factors/ social determinants
Health Promotion*	1. Outline the role of media in Health Promotion 2. Apply the disease prevention approach 3. Familiarity with health promotion policies and strategies 4. Plan health promotion based on research 5. Generate information to counteract industry (nutrition, tobacco, alcohol) 6. Design materials 7. Engage media
**Monitoring and Evaluation (M&E)**	1. Use M&E data to support decision making, advocacy 2. Design indicators for M&E public health programs, interventions and regulations 3. Monitor public health policy, interventions using input-process-outcome (impact) indicators 4. Evaluate public health policy, interventions using indicators 5. Conduct stakeholder interviews to evaluate 6. Determine community diagnoses to identify health challenges 7. Develop plan to address health challenges
Health Policy*	1. Assess policy options to address health problems 2. Assess feasibility of policy/ program using appraisal of evidence 3. Develop context sensitive strategic policy 4. Develop context sensitive strategic plan 5. Implement health policy
Communication*	1. Written skills 2. Oral skills 3. Integrate contributions from stakeholders 4. Listening and responding to different audiences 5. Convey health information appropriately to a professional audience 6. Convey health information to the public 7. Contribute to advocacy of health policies
Context specific*	1. Familiarity with how social factors affect access, availability, acceptability and delivery of health services 2. Familiarity with how social inequalities produce health disparities 3. Incorporate social determinants to assess health needs and interventions 4. Familiarity with how health system affects quality of care and people’s quality of life
Community and intersectoral*	1. Engage community actors to promote health 2. Partner with diverse stakeholders
Planning and management*	1. Use of evidence for planning interventions 2. Assess if Primary Health Care principles are used for planning programs 3. Plan and implement interventions using Donabedian metrics 4. Assist in managing information systems 5. Use economic evaluation for prioritization
**Crisis management and Emergency preparedness**	1. Describe processes to identify agent and their epidemiologic characteristics 2. Conduct outbreak investigation – characterize population and exposure 3. Use surveillance systems to identify threats 4. Appraise health alerts to assess implications for local context
**Academic/research**	1. Conduct literature review for decision making 2. Interpret research findings and communicate these 3. Develop a research proposal that upholds good methods and ethics 4. Identify conflict of interest 5. Reference sources 6. Write grant to support research 7. Develop a research budget.
Leadership*	1. Describe attributes of good leader 2. Adapt to cope with changes, e.g., COVID-19 3. Apply team building skills 4. Apply conflict resolution skills 5. Apply human rights and equity principles in decision making

* Competency category used in Zwanniken et al.’s study [21]

We use three lenses to assess self-reported competencies of MPH graduates – the applicability to their work settings; whether competencies were attained or acquired from the MPH; and respondents’ confidence in applying or using competencies in practice. Each lens was scored using a tailored four-item Likert score. Applicability was scored from: 1 = not applicable to 4 = very applicable; Acquisition from MPH: 1 = not from MPH to 4 = MPH enabled substantially; and confidence from: 1 = not confident to 4 = very confident. The wording of the SAQ is shown in S1 File, the questionnaire. The scores were recoded, to yield a maximum of 3 for each item, giving a minimum score of 0 and a maximum of 21 for domains with 7 items. The Likert recoded scoring system for each competency domain is detailed in S1 File.

Their recommendations for improving MPH courses were elicited through a Likert scale rating the desirability of three items – practical experience, problem solving skills and mentorship, as well as through an open-ended question. The scale ranged from 1–5: 1 = strongly disagree; 2 = disagree; 3=neither disagree nor agree; 4 = agree; 5 = strongly disagree.

### Data collection and response rate

The recruitment of respondents is detailed elsewhere [18]. Following ethics approval from all participating universities, we attempted to contact 710 eligible graduates (2012–2016) from all eight universities, regardless of nationality and country of work. For five universities, graduates were emailed through REDCap, inviting participation, and this enabled tracking of non-responders. As three universities’ academic departments were not permitted to divulge identifiers of their graduates, those alumni were invited by academics or university administrators through emails which contained a link to the on-line survey. Recruitment for all institutions commenced on 04/05/2022 and closed on 10/08/2023, except for UWC which started after 18/05/2022.

As graduation and up-to-date contact details varied by institution, there was uncertainty as to when some graduated and if eligible graduates were successfully contacted (see figure in S2 File). The denominator for response rates was determined by counting those that we attempted to contact by email (n = 658), short messaging service or by phone. Those invited needed to complete the online consent form before completing the questionnaire, by clicking ‘yes’ or ‘no’. In the case of the latter, the survey terminated immediately. Respondents did not have to complete the questionnaire in one sitting and could return it using a unique link.

Patterns in missing competency survey data were explored. Reviewing the data revealed that the frequency of missing data increased as the questionnaire proceeded, as depicted in Fig 1, from 167 responses at the beginning of the competencies section to 125 at the end, signifying attrition of 25%. Fig 1 shows that the extent of missing data was relatively consistent across each lens and domain, indicating comparable levels of questionnaire completeness within each domain. Missingness was, therefore, highly correlated across lenses for all competencies and domains. We determined that missing data were not related to hidden patterns or variables using Little’s Missing Completely At Random (MCAR) test by testing one lens for all 58 competencies in the 11 domains, which was not significant (p = 0.74). This “means that the missing data was a random sample of the complete data”, and could be considered ignorable [25]. Consequently, missing responses were not imputed, and likelihood-based methods were not used to generate values for the missing data. Scores were therefore calculated only for completed items, with the sample size reported for each variable analyzed.

**Fig 1:**
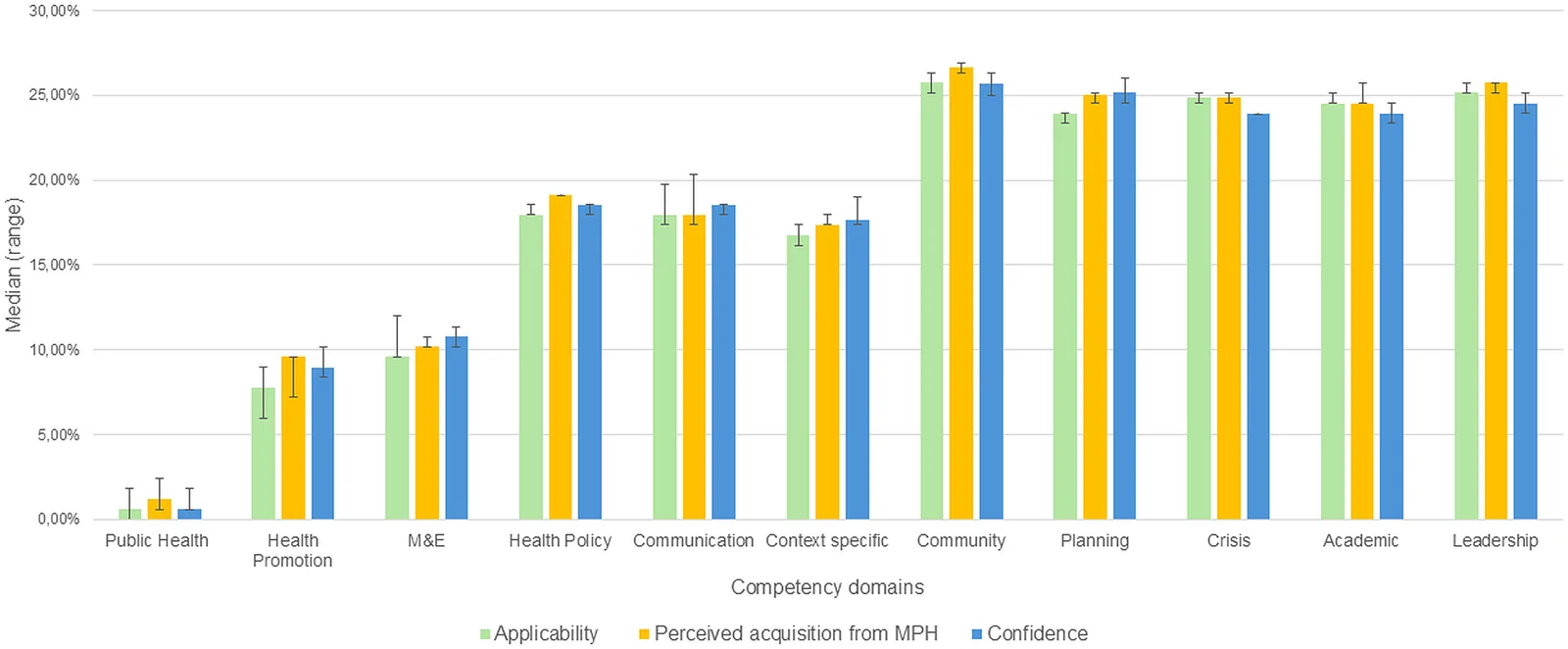
Median missingness of data by competency domain.

### Data storage and analysis

The REDCap application enabled encrypted data storage. No identifiers were captured, and the anonymized dataset was analyzed using STATA 13. Data was cleaned by removing respondents who graduated before 2012 and those graduating after 2017. We combined the responses of SMU and UL graduates as they were one institution from 2005 until April 2015.

### Quantitative data analysis

Descriptive analyses were undertaken for variables derived from closed-ended questions. Summary statistics were calculated for variables, including competency scores, with appropriate measures of central tendency for normal and non-normally distributed data, and 95% confidence intervals (CI) for proportions. T-tests and χ-squared tests compared means and proportions between groups.

As the Likert scores were essentially ordinal, competency scores for each respondent were calculated for each item in the 11 competency domains and totaled. All items were weighed equally. Totals were calculated for all three lenses – their applicability to work, extent they were perceived to be obtained from MPH and graduates’ confidence in their use.

Shapiro-Wilk tests were performed on all composite competency data – the 11 domains through the three lenses, which demonstrated that 31 of the 33 variables were not normally distributed (p < 0.05), as is detailed in Supplementary file 2. Consequently, median and interquartile ranges (IQR) were calculated, which is appropriate for ordinal data [26]. To make scores interpretable, comparable and actionable for a range of stakeholders, they were converted into percentages, by dividing the median and IQR score by the maximum possible score. This yielded 33 median score percentages (and IQRs) for the 11 domains through the three lenses. The count of scores for ‘not being applicable’ were analyzed separately. Unless otherwise stated, all averages given are medians. For each competency domain, correlations across the three lenses were assessed using Spearman rho (ρ) coefficients as we hypothesized that perceived competency scores across the three lenses were correlated:- confidence in exercising a skills set may be correlated with their use at work and/or perceived to be acquired from the MPH, and use at work may correlate with perception of being acquired from the MPH, and interpreted as per Dancey and Reidy [27].

The reliability of each domain metric for all three lenses was checked by calculating Cronbach alphas for each competency in the 11 domains, which is given in S2 File. All domains across all three lenses scored between 0.72 and 0.95 except for the community and intersectoral domain, where respondents’ confidence and their perception that their competence was derived from the MPH scored below 0.7. This domain enquired about only two skills. Over 0.70 is considered to be a benchmark value for a reliable measure with > 0.9 indicating excellent consistency [28]. A low number of questions can yield a low value of alpha, such as was the case for community and intersectoral competence [29].

Recommendations to improve the MPH program, ascertained through three closed ended questions, used Likert scales – of strongly disagree = 1; disagree = 2; equivocal = 3; Agree = 4; and strongly agree = 5 and the proportion of respondents’ ratings were calculated.

### Qualitative data analysis

All free comments for recommendations for MPH programs were coded manually, and grouped into themes by the first author. Responses were largely an elaboration of rated scores. Consequently, they gave deeper insight into what the scores were based on. Quotes illustrating themes are given, which substantiate scores. The country, age at commencement of the MPH and gender of respondents accompany the quotes.

### Ethical considerations

The study was first approved by the University of Cape Town’s Human Research Ethics Committee (HREC: 630/2018) and then by the participating institutions’ Ethics Committees, except for SMU which was combined with UL over the period which graduates were enrolled in MPH programs. The other approving Ethics Committees, all registered with the South African National Health Research Ethics Council, were UKZN Humanities & Social Sciences Research Ethics Committee (HSSREC/00002329/2021), UL Research Ethics Committee (TREC/372/2020), UP Faculty of Health Sciences Research Ethics Committee (795/2020); UWC Humanities and Social Sciences Research Ethics Committee (UWCRP165861) and Wits Human Research Ethics Committee (Medical) (M202256). For three universities, as required by Ethics Committees, recruitment was conducted by local academics. Contact email addresses were obtained for respondents requesting the survey findings, and these identifiers were subsequently removed from the data and stored separately. Respondents were given information about the survey; that they need not complete all sections; and could withdraw with no consequence; and could proceed only after agreeing to participation. We assured respondents that all information provided by them would be used only for the purpose of the study. Investigators could not identify non-responders, and automated reminders were sent to non-responders by REDCap. The identities of the participating universities are not given in the findings.

## Results

Respondents were alumni from seven universities (as SMU is reported with UL) that graduated with MPHs between 2012 and 2016. The response rate was 36.0% overall, but varied by institution, ranging from 18.1% to 70.3% (see Supplementary file 2 for figures). Graduates were mid-career professionals, with a median age of 35 (IQR: 29–42) on entry to the MPH, an average of 9 years of work experience prior to starting the MPH, and there were marginally larger proportions of females (57%) as seen in Fig 2. At the time of the survey, respondents were on average 46 years old (median; IQR: 41–53 years). Most were South African (53%) or from the rest of Africa (44%).

**Fig 2:**
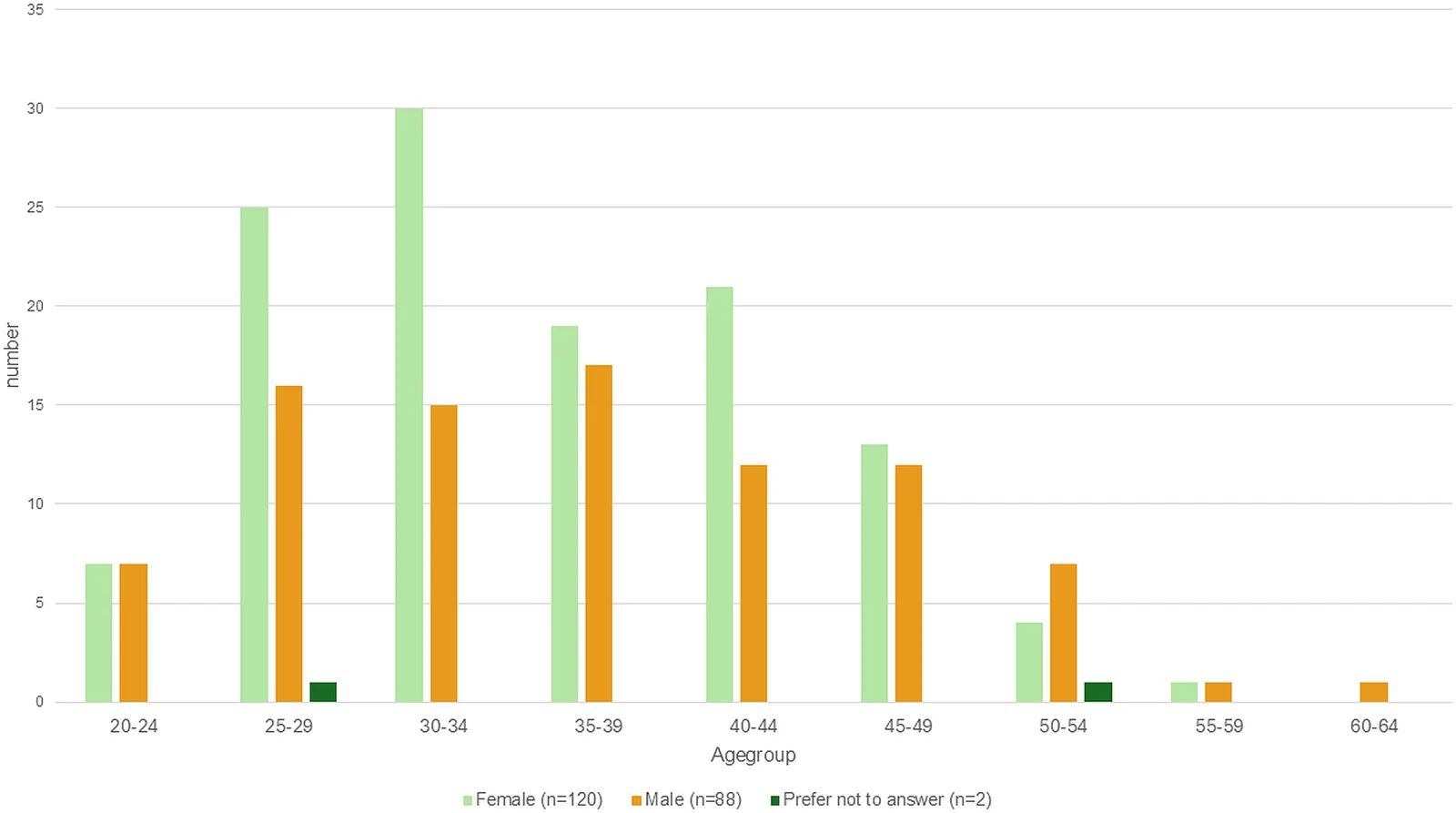
Baseline characteristics of respondents embarking on the MPH (n = 208).

### Useful acquired competencies and self-assessed proficiency

Respondents reported that they found most competencies applicable to their work, perceived they acquired them from the MPH and were confident using them. Overall calculated median scores for competencies through these three lenses are depicted in Fig 3 below. The content of items probed are given in Table 1.

**Fig 3:**
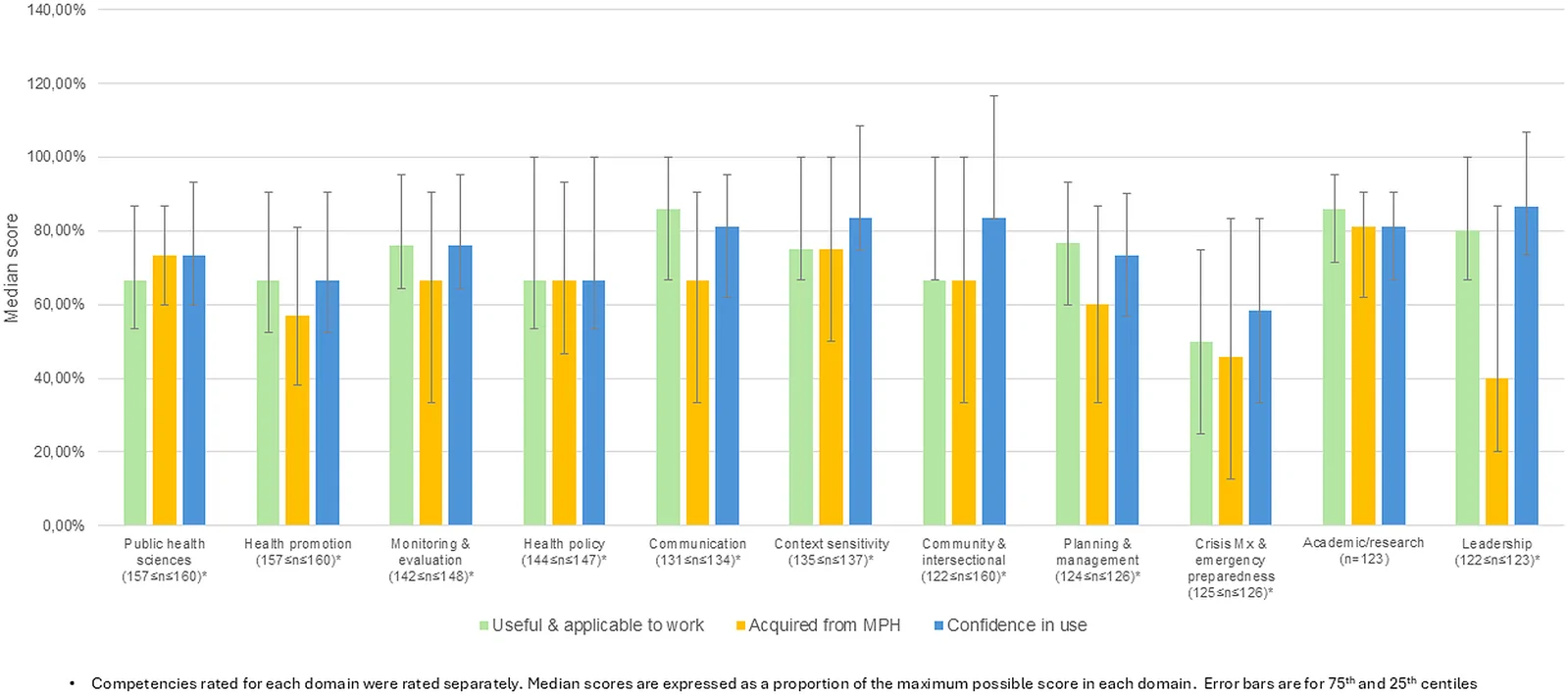
Self-reported competency scores by three lenses.

As seen in Fig 3, the highest scores for competencies being perceived to be useful at work were the communication and academic skills (86%; CI:71–95%), followed by leadership skills (80%; CI:67–100%). On average, over two-thirds of the other competency items were perceived to be applicable to respondents’ work, except for crisis management, where on average half of the four skills in the domain were seen to be applicable to their work. Similarly, 81% of academic competencies and over 60% of the other competencies were rated as being obtained from the MPH except for health promotion (57%; IQR: 38–81%), crisis management (46%; IQR: 13–83%) and leadership (40%; IQR: 20–87%) domains. Nonetheless, besides the crisis management domain, respondents rated their confidence as more than 67% in all competency domains.

As seen in Table 2, the scores for being applicable to respondents’ work, perceived to be obtained from the MPH and confidence in use, were mostly correlated and not independent (p = 0.00). The exceptions are in academic and leadership domains where confidence in using the skills and obtaining them from the MPH were weakly correlated (0.40 > ρ ≥ 0.1).

**Table 2:** Spearman correlations (rho = ρ), comparing the lenses – being applicable to job, obtained from MPH and confidence – for 11 self-perceived competency domains*.

	*Applicable to work* compared to confidence	Applicable to work compared to *obtained from MPH*	‘*Confidence*’ compared to ‘*from MPH’*
**Public Health**	0.77	0.64	0.74
**Health Promotion**	0.64	0.76	0.62
**Monitoring and Evaluation**	1.00	0.53	0.53
**Health Policy**	0.74	0.68	0.77
**Communication**	0.72	0.62	0.66
**Context & Intersectoral**	0.67	0.60	0.79
**Community**	0.85	0.56	0.64
**Planning & Management**	0.68	0.48	0.66
**Crisis Management**	0.64	0.77	0.63
**Academic**	0.61	0.50	0.37
**Leadership**	0.55	0.60	0.40
* p = 0.00 for all rho’s calculated		very strong	r_s_=1
		strong	0.90 > ρ ≥ 0.70
		moderate	0.70 > ρ ≥ 0.40
		weak	0.40 > ρ ≥ 0.1

### Competencies not needed, not acquired from MPH or unused

As given in Table 3, the proportions of respondents who perceived they did not require specific competencies for their current work, did not acquire them from the MPH or were not confident that they could use these competencies varied widely. For example, 19.9% did not use crisis management and emergency preparedness skills, while all used academic/research competencies. On the other hand, larger proportions perceived that they did not obtain leadership (26.6%) or crisis management skills (31.3%) from the MPH, compared to 3.2% and 6.7% who did not obtain research and public health skills respectively. Overall, small proportions (<6%) were not confident in all competency domains except for health promotion (13,1%) and crisis management (19.3%). Scores by each competency item in the 11 domains through the three lenses are given in S2 File.

**Table 3:** Respondents who reported that competencies were irrelevant to their work, *not* obtained from MPH and *not* confident in application*.

	Not applicable to work		Not obtained from the MPH		Not confident in application	
	Median % (IQR)		Median % (IQR)		Median % (IQR)	
Public Health science skills	7.2	(4.2-7.9)	6.7	(3.0-8.4)	3.6	(1.8-4.9)
Health Promotion competencies	5.2	(4.5-8.6)	11.3	(10.6-17.1)	13.1	(7.8-16.0)
Monitoring & Evaluation	4.7	(4.0-6.7)	14.1	(12.7-14.7)	5.4	(3.4-6.1)
Health Policy	10.2	(8.8-10.3)	7.4	(6.7-7.4)	5.1	(5.1-5.9)
Communication	2.9	(1.5-3.7)	11.0	(9.8-13.0)	1.5	(0.7-2.9)
Context sensitive	4.3	(4.1-6.4)	5.8	(5.1-7.3)	2.2	(1.8-2.5)
Community & intersectional	5.2	(4.1-6.4)	11.0	(9.8-12.3)	3.2	(2.4-4.1)
Planning & management (Mx)	10.3	(10.2-11.0)	12.8	(12.7-16.8)	3.2	(2.4-4.0)
Crisis Mx & Emerg preparedness	19.9	(15.5-24.7)	31.3	(30.0-33.1)	19.3	(16.1-22.4)
Academic/research	0.0	(0.0-4.0)	3.2	(0.8-15.9)	3.9	(1.6-6.3)
Leadership	2.4	(1.6-2.4)	26.6	(21.0-28.8)	4.7	(3.2-4.8)

* Median percentage of respondents, rating competencies were not applicable, useful or obtained through the MPH

### The eleven competencies

Respondents’ self-perceived competencies in each domain through the lenses – the correlations between usefulness, being acquired from the MPH and confidence in proficiency, varied by domain.

#### Public health sciences, comprising five items.

Most respondents (92.8%) rated public health skills as being important to their work. They rated that on average (mean and median), over two thirds of public health competencies were used at work (67%; IQR: 53–87%), and perceived that almost three-quarters (73%; IQR: 60–86%) of these were obtained from the MPH. Furthermore, they felt confident about using 73% (IQR: 60–87%) of competencies. Confidence in using public health skills was strongly correlated (p = 0.001) with perceptions of having acquired them during the MPH and finding them useful at work. However, their perceived acquisition of these skills from the MPH was moderately correlated with their applicability in the workplace (Table 2), suggesting that they viewed that taught skills were moderately useful.

However, from Table 3, a median of 7.2% of respondents reported that public health sciences competencies overall were not be applicable to their work contexts. They were not rated as being obtained from the MPH for 6.7% and 3.6% were not confident using these. This was influenced by the high proportions of respondents (>13%) who reported that environmental health skills, item 2 under public health sciences (see S2 File), were not applicable to their work (18.3%); 16.6% felt they were not obtained from the MPH; and 13.9% were not confident in using these skills.

#### Health promotion, comprising seven items.

The vast majority (94.8%) of respondents rated that health promotion competencies were used in their work, but only 57% perceived they obtained the competency from the MPH. This gap suggests that for some, health promotion skills were obtained elsewhere. On average, two thirds of health promotion skills were used at work (67%; IQR: 52–90%) and respondents were also confident in using two-thirds (67%; IQR 48–86%) of these skills. Perceptions about obtaining these skills from the MPH were moderately associated with applicability of health promotion in work settings (p = 0.00), suggesting some acquisition of these skills from the MPH. Their confidence in using these skills was also moderately linked to respondents’ perceptions that they acquired health promotion skills during the MPH (p = 0.00), signifying that taught skills were useful.

From Table 3, on average, a small proportion of respondents (5.2%) reported that health promotion skills were not relevant to their work settings. A minority, 11.3%, perceived that they did not obtain these from their MPH, and 13.1% were not confident they had health promotion competencies. There was one item – engaging the media about health promotion activities (item 7), where 11.8% said this was not applicable to their work; 17.1% perceived they obtained these from the MPH; and 18.4% were not confident using the health promotion skills.

#### Monitoring and evaluation (M&E), comprising seven items.

Around 95% of respondents rated M&E competencies as being important for their work. They used and were confident using three quarters of these skills (76%; IQR: 53–100%). Two-thirds of these skills (67%; IQR: 33–90%) were perceived to be obtained through the MPH. Use of these skills at work was moderately correlated to respondents’ reporting they obtained them from the MPH (p = 0.00) suggesting that M&E curricular material was useful. In addition, they used M&E skills confidently, demonstrated by the perfect correlation between the use of skills at work and their confidence in using the skills (p = 0.00).

As seen in Table 3, while small proportions (4.7%) reported they did not need M&E skills in their work and were not confident they had these skills, a higher proportion (14.1%) reported they felt they did not acquire these from the MPH. This implies that some respondents may have obtained M&E skills elsewhere.

#### Health policy, comprising five items.

Around 90% of respondents reported needing these competencies at work. These respondents used two-thirds (67%; IQR: 53–100%) of these competencies at work, perceived that they obtained them from the MPH (67%; IQR: 47–93%) and felt confident using them (67%; IQR: 53–100%;). Feeling confident using the skills was strongly correlated with respondents’ using them at work (p = 0.00) and perceiving that they were obtained from the MPH (p = 0.00), which suggests that respondents confidently utilized policy skills obtained from the MPH.

However, as seen in Table 3, 10.2%, reported not needing the identified policy competencies overall, but smaller proportions – 7.4% perceived that they did not obtain these from the MPH and 5.1% were not confident using these skills. Between 10% and 12% of respondents did not need three specific skills – the appraisal of evidence to ascertain the feasibility of policy implementation, policy making for specific contexts and policy implementation, given in S2 File.

#### Communication, comprising seven items.

Nearly all respondents (97.1%) rated communication competencies as being important to their work. They rated that 86% (IQR: 67–100%) of communication competencies were core to their work and they felt confident in 81% (IQR: 67–90%) of these skills (Fig 3). These two scores were strongly correlated (p = 0.00). However, respondents perceived that they obtained fewer communication skills from the MPH (67%; IQR: 33–90%). Both the use of these competencies and their confidence using communication skills were only moderately correlated with their perception about being obtained from the MPH (p = 0.00).

Table 3 shows that while small proportions reported not needing (2.9%) and not being confident using communication competencies (1.5%), more than 10% believed they did not obtain these from the MPH, implying that for some, these were obtained elsewhere.

#### Context specific competence, comprising four items.

Most respondents (95.7%) rated that context specific skills were applicable to their work setting. They rated that 75% (IQR: 67–100%) of these competencies were needed at work and they were confident using a higher proportion of skills – 83% (IQR: 67%−100%) (Fig 3). Their confidence in, and use at work, of these skills were strongly correlated (p = 0.00). Obtaining these skills from the MPH was strongly correlated with their confidence using these skills (p = 0.00), which suggests that contextual skills that they believed were acquired from the MPH, were used with confidence. However, their rating that skills acquired from the MPH were moderately correlated with use at work (p = 0.00), suggests that there may be contextual competency gaps in the MPH.

From Table 3, small proportions did not need context specific skills (4.3%) or lacked confidence using them (2.2%), but a marginally larger proportion (5.8%) believed they did not obtain these from the MPH.

#### Community and intersectional competencies, comprising two items.

Most respondents (94.8%) rated community and intersectional skills as being important in their work settings. Around two-thirds of these skills (67%; IQR 67–100%) were needed by respondents and were viewed as being obtained from the MPH (IQR: 33–100%), but these proportions were only moderately correlated (p = 0.00). Respondents were confident in using 83% of these skills (IQR: 67%−100%), and confidence was strongly correlated with use at work (p = 0.00), suggesting these skills were used with confidence.

However, from Table 3, small proportions (5.2%) did not need community and intersectional competencies, and a similar percentage (3.2%) were not confident using these but 11.0% reported they did not get them from the MPH.

#### Planning and management competence, comprising five items.

Almost 90% needed planning and management skills in their work settings and they rated that 77% (IQR: 60–93%) of these skills were important in their work settings (Fig 3). However, they saw that they only obtained an average of 60% from the MPH (IQR: 33–87%), suggesting an unmet need. Respondents were confident in 73% of planning and management skills (IQR: 60–93%). Their confidence was moderately correlated with the usefulness of these skills at work (p = 0.00), as well as being obtained from the MPH (p = 0.00).

Conversely, Table 3 shows that a median of 12.8% did not require planning and management skills, yet only 3.2% were not confident using these competencies, and 12.8% reported that they were not acquired in the MPH. These proportions were affected by one item – their skills in managing and evaluating health information systems (item 5, Table 1), which was not needed by 15.7% of respondents, not obtained from the MPH by 24.8% and 9.6% were not confident they possessed this competence.

#### Crisis management competence, comprising four items.

Only 80.1% of respondents rated crisis management skills as being important in their work settings. As shown in Fig 3, this is the domain that respondents rated that they obtained the lowest proportion of skills from the MPH (46%; IQR: 13–83%). They used on average half (50%; IQR: 25–75%) of these skills and were confident in 58% of skills (IQR: 33%−83%), which were moderately correlated (p = 0.00). The scores for perceiving they obtained skills from the MPH and use at work were strongly correlated (p = 0.00) which suggests that their learnt crisis management skills were used. A sense of them being obtained from the MPH was only moderately correlated with confidence (p = 0.00), suggesting that some confidence in crisis management came from other experiences.

On the other hand, Table 3 shows that almost 20% of respondents perceived they did not require and were not confident in using crisis management skills. Additionally, over 30% perceived that they did not obtain these from the MPH. This suggests an unmet need as well as that many respondents may not have been directly involved in managing the COVID-19 pandemic, where these skills would have been used.

#### Academic and research skills, comprising seven items.

Respondents needed, perceived they were obtained from the MPH and were confident in 80% or more of academic skills –86% (IQR: 71–95%); 81% (IQR: 62–90%) and 81% (IQR:43–100%) respectively. Despite these proportions being similar, their confidence in research skills was weakly correlated to their sense that these were obtained from the MPH (p = 0.00) (Table 2). This is due to the 20% (CI: 14.4–28.7%) of respondents who rated that they did not acquire research budgeting skills from the MPH and the 10% (CI: 5.9–16.8%) who were not confident with budgeting (see S2 File). This suggests that these skills, needed by 95% of respondents, were obtained elsewhere.

While academic and research skills were needed by all respondents, a small proportion (3.2%) perceived that these were not obtained through the MPH and 3.9% of respondents were not confident using these skills (Table 3). The skill set that disproportionately more respondents (10.2%) felt least confident possessing was ‘developing a research budget’, which while being applicable to work for 96.0%, was felt to not have been obtained from the MPH by 20.6% (item 7, Table 1).

#### Leadership competence, comprising five items.

Despite the MPH being seen as the source of 40% of leadership skills (IQR: 20–87%), almost all respondents (97.6%) believed they required leadership skills at work, and 80% (IQR: 67–100%) of leadership skills were used. While being important at work, these skills were only moderately correlated with being obtained from the MPH (p = 0.00) and with respondents’ confidence (p = 0.00) (Table 2). Respondents were confident using 87% (IQR: 67–100%) of the skills identified, but their confidence was only moderately correlated with their perception that these were obtained from the MPH (p = 0.00), suggesting some skills were obtained elsewhere.

As shown in Table 3, a small proportion (2.4%) of respondents felt that they did not require the named leadership skills in their workplace, and 4.7% were not confident using this range of skills. The MPH was seen as not being the source of these skills for a sizeable proportion (26.6%) of respondents, which suggests that for some, these were acquired elsewhere.

### Improving MPH programs

Respondents rated improvements to the MPH program in three areas. Fig 4 shows that over 65% and 90% of respondents respectively agreed that mentorship programs and practical exposures would be useful additions to MPH programs. Most (>90%) also agreed that problem solving skills would be a useful additional skill.

**Fig 4:**
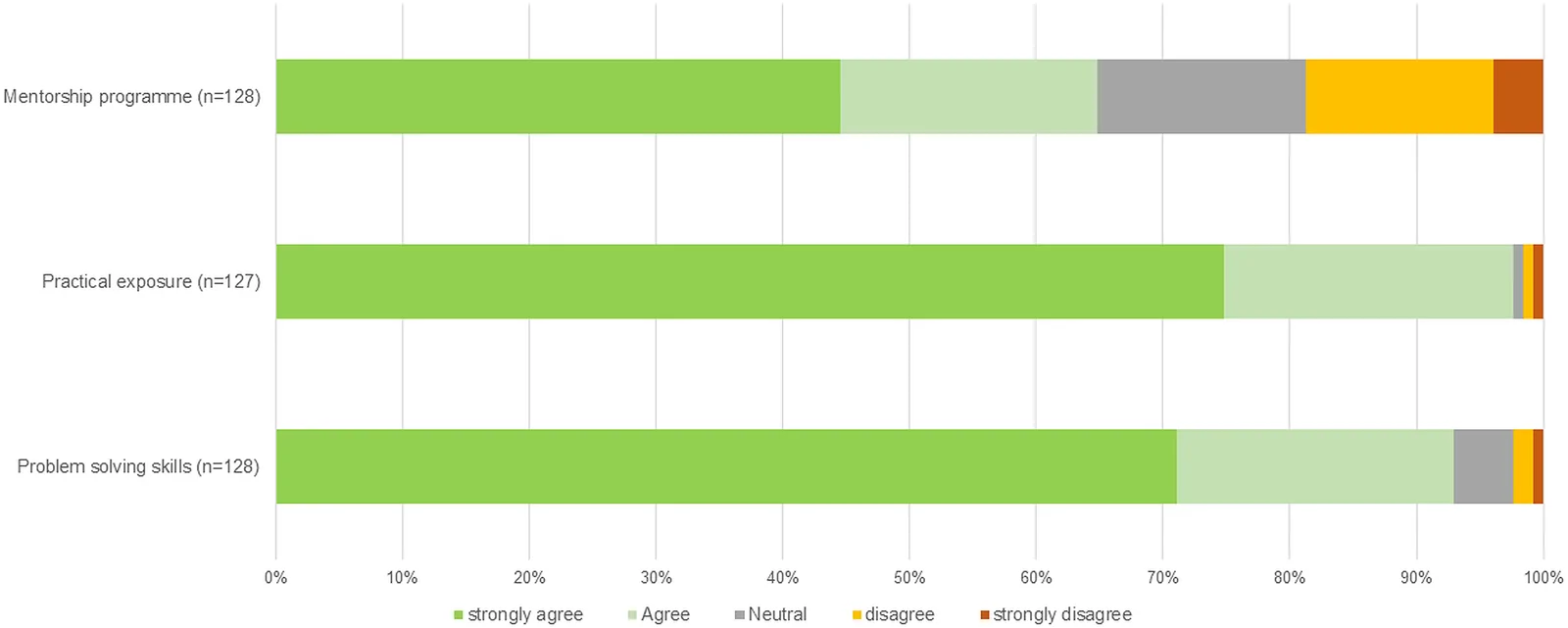
Respondents’ rating of identified additions to MPH programs.

Despite respondents being appreciative of MPH programs and believing that the degree was highly regarded and opened work opportunities [18,19], 36 respondents suggested strategies that universities could adopt to make learning experiences and competencies acquired more applicable to public health work. These respondents did not differ by South African origin, age, sex and to all respondents (South African = 46.3% compared to 53.2% overall (p = 0.364); mean age at commencement of MPH = 38.1 years compared to 36.0 years overall p = 0.26; females = 52% compared to 57% overall p = 0.61). However, they differed by institution. Respondents from one institution that recruited its own alumni, contributed 46.3% of respondents giving free comments while it comprised 29.8% of the sample (p = 0.023). Respondents’ comments complement the proposed additions rated in Fig 4, and center on three themes – the MPH course content, program organization/structure and faculty needing to further graduates’ competencies and work, given in Fig 5.

**Fig 5:**
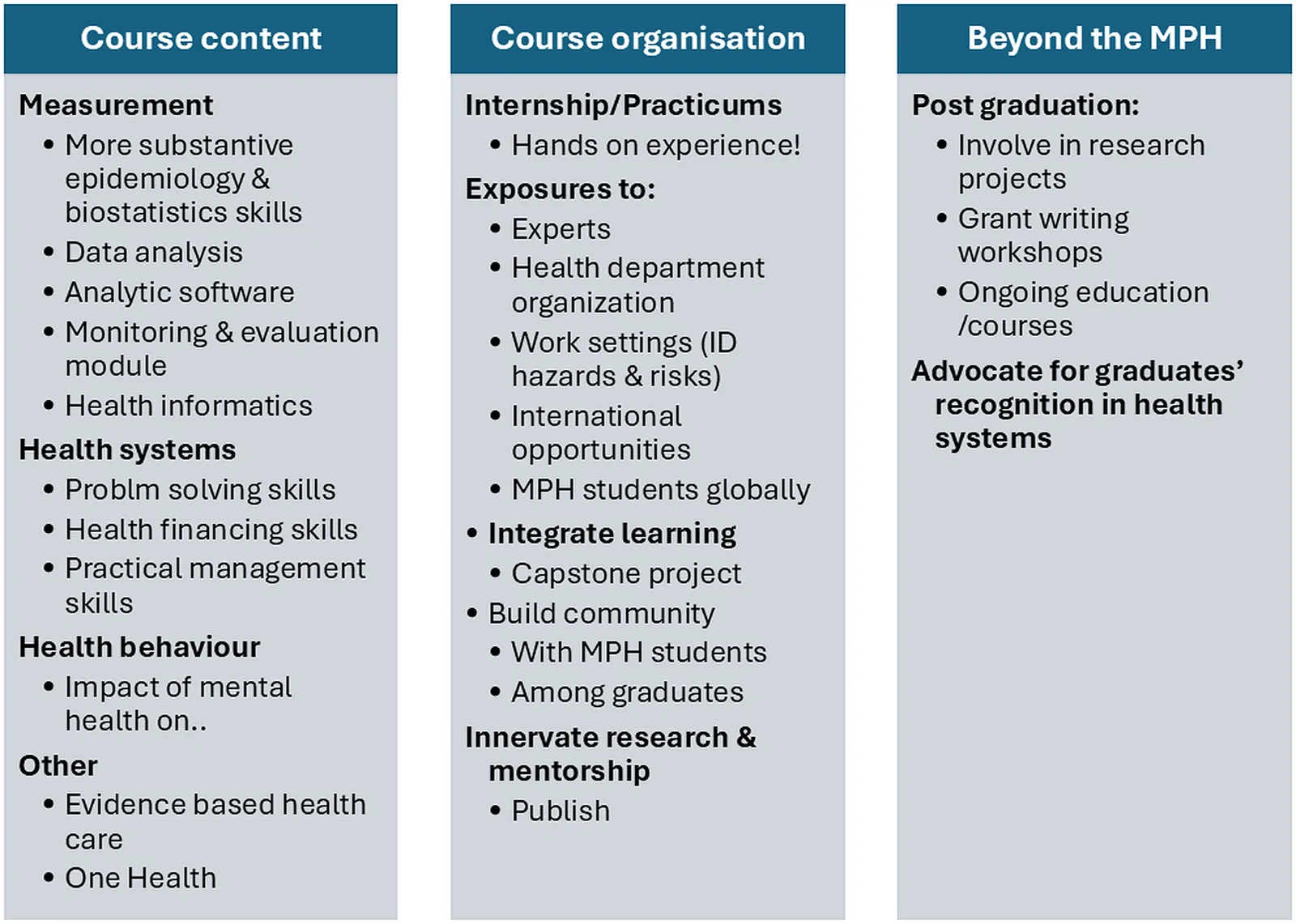
Respondents’ recommendations for improvements to MPH programs.

#### Course content.

Respondents raised the importance of epidemiology and biostatistics skills for public health practice, and some felt this training could be enhanced, particularly in the management and analysis of data, learning data software skills and data presentation. Others highlighted the importance of M&E skills as these are core to public health work.

Public health is anchored on two pillars of Biostatistics and Epidemiology. Therefore, MPH can be improved through the inclusion of fully fledged courses in Biostatistics and Epidemiology as opposed having them under Measuring Disease and Health. (Zambia, 50, M)

I … propose the importance of providing strong data analysis module for MPH students as it is the core in the development of their career as both researchers or [in] programs. (South Africa, 27, M)

Training on data analysis software like [ ] and data presentation applications like [ ]. (Kenya,26, M)

Health system skills that were proposed included enabling students to problem-solve real problems encountered in management and health delivery contexts which should include decision-making for resource allocation. This would enable graduates to obtain practical management skills.

What I would encourage is to have real time issues, when teaching management, problem solving issues and decision making. Have some real time attachments to organizations where students can come back equipped in the area. (Zambia, 50, F)

One highlighted including aspects of health promotion such as the impact of mental health on health decision-making and health seeking behaviour as well as communication skills to impact on health behaviour.

More learning on health behaviour and communication with an element of understanding the impact of mental health (South Africa, 48, F)

A critical public health skill set raised was the assessment of evidence for decision-making. Some argued that MPH graduates, who contribute to developing guidelines for decision-making, should take a course in evidence-based health care.

A push … to upskill students in evidence-based health care and contributing towards the evidence eco-system. Be it reading and understanding systematic reviews... Conducting systematic reviews... Furthermore, contributing toward guideline development. (South Africa, 20, M)

One graduate with a veterinary background argued that the concept of ‘One Health*’* should be central to public health learning, as the environment affects both human and animal health. As zoonoses are increasingly recognised as a source of ill-health, they proposed that animal health professionals should be part of teaching programmes.

I recommend for the inclusion of multidisciplinary [animal health practitioners] skill if the program is to achieve.. [the] One Health Concept. Decision makers must integrate this multidisciplinary skill. (South Africa, 39, M)

#### Course organization.

Innovative changes were proposed that would enable students to engage with, obtain experience of and reflect on public health work in real life settings. Many respondents suggested including internships and practicals, where they deepened competencies, through applying skills in projects identified by hosts.

Have some real time attachments to organizations where students can come back equipped in the area. (Zambia, 50, F)

Provide students with practical training opportunities, such as internships or fieldwork, to gain hands-on experience in public health. This will help students to develop practical skills that they can apply in their careers. (Zimbabwe, 32, F)

Assign them to practical work through contextual projects. (Tanzania, 47, M)

Others added that experiential learning would enable better understanding of public health, its scope and opportunities. Suggested exposures included engagement with local public health departments and officials to obtain an understanding of their work and challenges; with industry to develop critical skills such as identifying hazards and risks to population health; and with the global public health community, including students, alumni and experts.

Offer students opportunities to visit the industries where the drinks, waters or foods are made or manipulated so that they can detect the public health issues in those sectors. (Congo, 53, M)

Ensure MPH alumni are invited to participate in sessions during the school term to offer [their] perspectives … from the field, post MPH. (Uganda, 36, M)

Students should be sent to all the relevant places to be trained by experts in the field before choosing a research project. (Nigeria, 32, F)

These respondents suggested that learning from academic and practical exposures should be integrated. To enable linking theory and practice, one suggested they could complete an overall assignment, applying their diverse set of skills to a public health scenario – a capstone project. Others suggested that students be exposed to intervention strategies, including implementation science, that address public health issues, where theory and interventions are systematically, iteratively applied and assessed.

More practical work linking implementation and academic. (South Africa, 27, M)

Assign them to practical work through contextual projects… and after … modules to write a capstone assignment. (Tanzania, 47, M)

Teach more about public health intervention strategies to students. (Malawi, 41, F)

Many suggested that a community of public health practitioners – both students and professionals – should be built. This would expand students’ perspectives and promote experiential learning.

Better sense of community between same cohort of students. (USA, 25, M)

More interactions with students and leaders outside of the continent to broaden one’s perspective. (South Africa, 32, F)

Research reports are required in South African MPH programs. Some respondents raised that research skills require more nuanced attention. They highlighted the importance of support for weaker students; for mentorship; and access to funding to conduct meaningful studies. They advocated that mentorship would both assist students completing their projects and publishing research findings. They believed that publishing completed research would showcase scholarship from Africa and develop graduates’ critical skills and academic writing.

MPH students require more support from tutors especially in the area of research… Tutors should be more patient with the weaker students instead of focusing only on smart students. (Nigeria, 39, F)

I think students should be obligated to publish a peer-reviewed article after successfully completing their MPH course as an incentive to increase publications from African authors. (Mozambique, 27, M)

Constant support and mentorship towards the end of the MPH degree just to assist graduates to publish or just a follow up in particular long-distance students. (South Africa, 31, F)

#### Furthering graduates’ competence and work.

Some added that support for students and mentorship should continue post-graduation, and institutions should keep contact with alumni. This would enable students to publish their research findings – a key academic competence, and be involved with university-based research. Suggestions of universities convening courses to update alumni’s skills and follow-on professional doctorates were also made.

The student [should] continue a “mentorship” relationship with research supervisor at least for one year, where possible; assist/encourage students to publish their research project. Each MPH student SHOULD be able to prepare a complete grant application by end of MPH studies. (Namibia, 41, F)

Organise get together meetings… ask them to publish their research, involve them in projects from their universities. (Tanzania, 47, M)

I think MPH graduates should have [access to] post-MPH modules offered at universities in topics relevant to the time... The landscape of public health is ever-changing. I don’t think one is ever done learning and adapting in public health. (South Africa, 48, F).

Offering opportunities post MPH for PhD … Develop DrPH (Non Academic). (South Africa, 51, M)

Some respondents highlighted inequities in the health system for MPH graduates, identifying that medical doctors with MPHs are employed preferentially compared to other graduates. They recommended that a registering body for MPH graduates, recognised by employers, is required which could include specialisation areas that could make skills sets explicit.

Medical doctors with MPH are well recognized as opposed to other professionals… Lobby for all … to recognize all MPH students equally, regardless of their profession. A body governing all this is required. (Botswana, 47, F)

Despite me having an MPH, I cannot automatically apply for vacancies in the human health without prior registration with HPCSA. This is a hindrance for someone who has registered with veterinary council as an example. (South Africa, 39, M)

The areas of MPH specialisation should be carefully defined (Zambia, 50, M)

Some believed that prospective employers had limited understanding and appreciation of the range of skills that MPH graduates could bring to the health services. They motivated that universities need to engage and partner with the services in projects and identify what academics and their graduates could offer.

Engagement [with] the employers in understanding the role and mandate of public health. (Botswana, 47, F).

Better/improved interaction with national government and support/direct interventions, when necessary, such as COVID-19 response, listeriosis outbreak. Academic institutions are better placed for knowledge management and sharing and as part of training a collaborative health conference/platform between institutions, government, local and international NGOs implementing programmes would be beneficial to all. (Zimbabwe, 42, F)

## Discussion

Prior to and since the COVID-19 pandemic, much work has taken place to identify the competencies of the public health workforce, the most recent being the WHO public health road-mapping project [3]. In high income countries (HICs), professional bodies developed and refined competency frameworks for public health practice and included areas that incorporate attitudes and skills in addition to knowledge –so-called ‘cross-cutting’ competencies. These include the USA (2021) [30], Europe (2020) [31], Canada (2017) [32] and Australia (2016) [33]. Work on competencies for MPH programs has also been conducted in some LMICs. For example, in 2013, eight core competencies were identified for public health practice in India [34], and the task force mandated by the Ethiopian Public Health Association in 2014, formulated nine core public health competencies, including for MPH graduates [35]. In Latin America, the Pan-American Health Organization (PAHO) 2013 framework identified six competency domains [36], which shifted in 2020 to three ‘pillars’ of public health functions– ethics to address inequities, tackling social determinants, and assuring access to public health services [37]. While there are calls for programs to expand graduate public health training in “epidemiology, health informatics, and program leadership” in Africa [11], there are no regional sets of competencies agreed upon for Sub-Saharan Africa or national set in South Africa. Notwithstanding the broad range of public health practice work settings, we see this study as a part of the process towards determining competencies needed and acquired from South African MPH programs.

This research, the first known from South African and African universities with MPH programs, explored graduates’ self-perceived public health competencies, through three lenses – their use at work, their acquisition from the MPH, and respondents’ confidence in applying skills. These three lenses speak to Miller’s seminal work developed to determine clinical competence, the four-level Miller’s pyramid [38] – the base being ‘knowledge’, followed by ‘knows how’, then ‘shows how’ with the pinnacle being ‘does’ – the ability to confidently ‘do’. As these elements were gauged in this study, the three lenses investigated align with metrics used in health sciences education literature.

We based the tool used on the validated set of competencies advocated for the evaluation and design of programs in LMICs [20], and quantified a comprehensive framework of 11 competency domains. Besides community competence, all ten other competencies through all three lenses were found to be reliable. We also elicited recommendations for improving MPH programs.

The respondent cohort completed the MPH during the second year of the COVID-19 pandemic. The pandemic required many public health professionals to step-up, multitask, and take on additional roles, and manage crises. COVID-19 was a wakeup call in many country settings – that public health professionals should be involved in evidence-based decision-making in crises [39], and the importance of including crisis management skills in curricula [40].

The vast majority of this mid-career set of African MPH graduates from multiple South African universities, the majority of whom are from South Africa, used many of the 11 public health competencies at work, which were largely obtained from the MPH, and most were confident using them. Except for academic and leadership competencies, the three lenses were strongly or moderately correlated, demonstrating that competencies were largely seen to be acquired. MPH graduate studies conducted in LMICs a decade ago and more recently in Israel, demonstrate that learnt competencies were applied at work [21,41].

Besides crisis management (not needed by 20%), over 90% rated that they needed all other ten competency domains at work. Over 85% needed skills in all domain items. In particular, the importance of academic and leadership competencies was highlighted and over 97% used them at work, which are key work roles that were also identified by MD/MPH graduates in the USA [42]. Competencies in critical analysis, leadership and research were also identified as important for work by international alumni from a UK public health masters course [43].

The contribution of the MPH to their skills is clear, as over 85% of competencies were perceived to be obtained from the MPH, except for leadership (40%) – as is reported elsewhere [18], crisis management (46%) and health promotion (57%), signifying that these courses may not have been taken by some respondents or the material was inadequately or not explicitly taught. Few of the MPH programs focus explicitly on health management, which explains the identified gaps in leadership and management training. To address the need for skilled health managers, many South African universities offer additional post graduate diplomas in health management.

Respondents overcame mismatches for crisis management and leadership. While crisis management, in time, may be integrated into curricula, the perception that health promotion competencies were inadequately derived from the MPH is concerning. It is firmly a core public health competence, is intrinsic to public health practice, and was rated by ASPHER as being their best success in Europe [44]. It may imply that health promotion courses were not followed by some respondents, the health promotional competencies scored are not what is taught, and curricula need to be re-examined to align with contemporary health promotion practice. As was found in this study, ASPHER also identified poorest success in preparedness for public health emergencies [44]. Despite this gap, public health professionals are needed to take on leadership roles in health systems, managing bottlenecks and crises [2,45]. Consideration must be given to how skills core to public health practice, such as monitoring and evaluation, crisis management and leadership can best be strengthened in MPH programs, preparing graduates to be fit for purpose.

Use of less didactic teaching methods to better prepare graduates for public health practice is highlighted in recent literature [46], a consideration also raised in respondents’ recommendations. Overwhelmingly the inclusion of exposures to the professional life of those working in public health practice, mentorship, networking and problem-solving skills in MPH programs were recommended, as were also recommended by young public health professionals in Europe [47]. This would require changes in course organization to accommodate recommended exposures such as engagement with global public health experts and MPH students elsewhere; public health practitioners in health departments; and fieldwork in environmental health. Such exposure may require students to engage online with experts and in forums. If well designed, these opportunities could also address gaps in institutional curricula due to inadequate local expertise and could facilitate learning. Importantly, experiences where students can develop and apply skills, integrate theory and practice through exposure to public health work settings would need to be accommodated in course work. Experiential learning, the process of experience, reflection, conceptualization of the experience and experimentation [48], facilitates deep learning and, with careful networking and mutually beneficial arrangements, could be embedded into South African MPH programs. Such learning is integral to the USA Council on Education for Public Health (CEPH) accredited programs – MPH students complete an integrative learning experience (ILE), with a written report, to demonstrate synthesis of competencies that is useful for students and community partners [49]. An evaluation of one such ILE, a Capstone project – which was proposed by a respondent, found that interpersonal skills were honed as well as technical skills in project management and practical research [49].

As was also highlighted by early-career family physicians and piloted in sub-Saharan Africa [50], many suggested mentorship in research to support completion and publication of research findings. More substantive skills in health measurement, evidence based health care, health systems, health behavior, and ‘One Health’, an area highlighted by the COVID-19 pandemic [51] were recommended. ‘One Health’, an area of education and practice has emerging competency domains [52] that clearly overlap with the MPH competency framework used and skills probed in this study. Additional skills in data analysis, software and M&E competencies were advocated and competence in data science has also been advocated for public health leaders [53]. Almost all used over three-quarters of the M&E competencies at work but fewer were felt to be obtained from the MPH. Attention has been given to M&E competencies and pedagogy in other LMICs [54] which could be examined and adapted in African settings.

Respondents made recommendations regarding registration of the MPH as a professional qualification, and they highlighted that universities should advocate for the recognition of the MPH and engage employers about the employment of graduates regardless of their original qualifications. Recognition of the MPH as a professional qualification is a complex project, which requires collaboration between a diverse sets of actors – the range of public health professionals, their professional organizations, training organizations, employers and certifying institutions together with health system policy makers and regulators [55]. As was reported in India, the absence of an accrediting institution does affect the quality of public health education [12]. In addition, as is reported in Ethiopia [56], competency frameworks will remain fragile without such institutionalization, which requires allocation of resources and stakeholder ownership [57]. However, the pandemic highlighted that professionalizing public health must become a priority. Spurred by the local public health association, this could be boldly led by Schools of Public Health who engage the range of stakeholders to review public health qualifications, appropriate competencies of graduates, the accreditation of teaching programs by a certifying institution, and the development of a register of professionals, which should assure employers of employee competence.

### Strengths and limitations

This national survey comprised alumni from all South African universities with MPH graduates between 2012 and 2016, a notable strength. Despite concerted institutional effort to encourage participation the 36.0% response rate, slightly below the 39.3% median response rate found in a metanalysis of online surveys [58], potentially limits its findings. Delays in executing the study due to COVID-19 resulted in participants having a longer post MPH work experience, extending the anticipated 4–8 years’ work experience to 6–10 years. This may have resulted in an information bias, with respondents recalling incorrectly what skills were obtained from the MPH. They may have also overestimated their acquired skills overall and those derived from the MPH, particularly as is reported elsewhere [19], the MPH was pivotal to them shifting careers away from direct patient care to public health careers. Importantly, the competency ratings are self-assessed, and respondents may not use the same yardstick. Poor ratings for crisis management skills may be the result of an Information bias, as respondents’ perceptions of their low competence in crisis management may be overestimated due to workloads due to COVID-19 that felt overwhelming. A study strength, however, is its timeliness factoring in the impact of the pandemic on the public health workforce and training.

As was their prerogative, many did not finish the survey. This may be due to its length despite not needing to complete it in one sitting. Nonetheless, the volume of missing data increased as the survey progressed, reducing the sample size for analysis. The presence of missing data was not due to other variables being probed. Reduced small sample sizes contributed to less precision in summary estimates and the wide confidence intervals found. Nonetheless, as reported elsewhere [18], compared to the overall source population, no systematic selection bias was found as respondents were equivalent for age, sex and of South African origin. However, the differences in response rates by institution is a selection bias that overestimates responses from more represented institutions. This is also true of the qualitative recommendations section where graduates from one institution are prominent. In addition, respondents may be more positive about the MPH and competencies acquired than non-responders.

These potential sources of bias, the limitations discussed may have implications for the validity and generalizability of the study findings. Mitigating against these weaknesses could be addressed through triangulating data from a qualitative study with 1−1 interviews among graduates, which was performed and is being prepared for publication. The overall mixed methods design is a strength, and the free comments give substance to quantitative findings. Other methods to triangulate data such as employer perspectives about the value and competencies of MPH graduates would be useful and could validate or contradict study findings.

The 53 competencies measured in the study may contain redundant items and we did not conduct factor analysis which could reduce the large number of variables measured into underlying factors that capture constructs. This was outside the scope of this study and further research to refine the tool is appropriate.

We used and adapted the competency framework, domains and dimensions from other research in LMICs [20], and did not build on the more recent WHO roadmap project that was developed after the COVID-19 pandemic. While there is alignment with many EPHFs – M&E, emergency management, leadership, health promotion, community engagement and research [3], further work is required to map our set of competencies to the EPHF domains and practice activities. We did not invite respondents to elaborate on perceptions and experiences of applying competencies, which are better elicited through qualitative research among MPH graduates. We also did not investigate whether these competencies were the intended outcomes of MPH programs or whether these were the key skills set required by employers, aspects which we recommend should be pursued.

## Conclusion

This study aligns with the WHO roadmap project [10] designed to embed the public health workforce into country health systems. It has generated useful information and contributes to understanding the competencies of MPH graduates from South African programs. It indicates what competencies are needed in professional practice in sub-Saharan Africa, what professionals feel confident using and what they believe is obtained from South African MPH programs.

Factoring in study findings into MPH program reviews is a first step towards generating core competencies in South African MPH programs, despite the absence of governance systems for public health programs, personnel and practice. Attention needs to be given to enhancing leadership, crisis management and health promotion competencies into existing courses, integrating them into assessments, as well as introducing practical experiences of public health work. Exposures to public health leaders, experts and organizations could fill gaps in skills such as problem-solving and project management skills, including budgeting, a highlighted gap. The production of a ‘fit for purpose’ graduate and workforce requires an alignment of education objectives with health workforce systems in country contexts to ensure a seamless transition from learning to employment.

## Supporting information

S1 File Participant information sheet, consent form and questionnaire used in survey. (DOCX)

S2 File Supplementary figures and tables. (DOCX)
