# Supplementary material for: Self-perceived competencies of Master of Public Health graduates and recommendations for programs in South Africa
Source: PLoS One. 2026 Oct 16;21(10):e0360789. doi: 10.1371/journal.pone.0360789 (PMC13674699; doi:10.1371/journal.pone.0360789)
Supplement: Supplementary file 1 [file pone.0360789.s001.docx]

Participant information sheet, consent form and questionnaire used in survey

1. Information sheet
2. Consent form
3. Perceived competency questions,
4. Recommendations for MPH programs
5. Scoring system

# 1. INFORMATION SHEET

## Introduction

We are a team of researchers, listed below, from different universities in South Africa that provide the Master of Public Health (MPH) degree programmes. We are conducting research on the perspectives of MPH graduates on the following:

- Reasons for embarking on an MPH,
- Perceived value and benefits,
- Career paths since MPH commencement;
- The application of their MPH learning in their work or personal lives
- The knowledge gained and skills learned as a result of the MPH
- Reflections on the role of public health during the Covid-19 disaster in South Africa.
- Their recommendation of revisions and changes to the MPH curriculum	taught at South African Universities and core competencies to be included

The research involves the completion of an electronic survey, which will take about 25 minutes of your time.

### Voluntary participation

We would like you to participate in the survey, and you are free to withdraw at any time should you wish. It will provide valuable information that could inform University and health policies in South Africa. However, your participation in this study is voluntary and there will be no negative consequences if you do not want to participate. The information obtained on MPH graduate career paths may assist the development of public health professionals’ career paths, particularly in the health systems of South Africa and other African countries as they strive to achieve the global goal of universal health coverage.

## Potential benefits and risks

There are no direct benefits, such as remuneration, for participating in the study. However, there are indirect benefits of the study. As this is the first multi-centre MPH study at South African universities, the study results could inform health workforce planning and training in South Africa, improve post-graduate selection and training programmes at the participating universities. Should you want, we will also provide you with feedback about the results.

We anticipate that there will be no risks or injury to you when participating in the study, and you will not be expected to cover any cost related to the study. Should you need data time for completing the study please let us know and we would gladly purchase data to cover this.

## Confidentiality

All the information collected will be kept strictly confidential. Study participants will all be assigned a code. These codes will only be known to members of the research team. We undertake that all information provided by you will be used only for the purpose of the study. Your identity and your individual choices will not be revealed or reported to others, or in any publication resulting from the study. Any other information that can potentially identify you in this study is being sought for follow-up purpose only and will not be used within the study. The aggregated results for each university group class and the entire group of all university MPH graduates will be published in peer reviewed journal articles.

## Ethical Approval

Ethical approval for this study has been obtained from each of the participating Universities.

### Questions

We will be happy to answer any question you have about this study. If you have any queries about the study, please contact the researcher at the institution where you trained or anyone from the list below.

| **Researcher** | **Institution** | **Contact details** |
| --- | --- | --- |
| **Dr V Zweigenthal** | University of Cape Town | [virginia.zweigenthal@uct.ac.za](mailto:virginia.zweigenthal@uct.ac.za) |
| **Dr T Dlungwane** | University of KwaZulu-Natal | [dlungwane@ukzn.ac.za](mailto:dlungwane@ukzn.ac.za) |
| **Prof L Rispel** | University of the Witwatersrand | [laetitia.rispel@wits.ac.za](mailto:laetitia.rispel@wits.ac.za) |
| **Ms N Schaay** | University of the Western Cape | [shaay@mweb.co.za](mailto:shaay@mweb.co.za) |
| **Prof T Tshitangano** | University of Venda | [takalani.Tshitangano@univen.ac.za](mailto:takalani.Tshitangano@univen.ac.za) |
| **Prof M Mokgatle** | Sefako Mokgatho University | [mathildah.mokgatle@smu.ac.za](mailto:mathildah.mokgatle@smu.ac.za) |
| **Prof F Matala** | University of Limpopo | [france.matlala@ul.ac.za](mailto:france.matlala@ul.ac.za) |
| **Dr SM Patrick** | University of Pretoria | [sean.patrick@up.ac.za](mailto:sean.patrick@up.ac.za) |

We will be happy to answer any question you have about this study. If you have any questions about your rights as a study participant, or questions or concerns about any aspect of the study, you may contact any of the relevant individuals listed in this information sheet.

2.	CONSENT

| I hereby give my consent to participate in this study. | Yes | No |
| --- | --- | --- |
| I understand I am taking part freely without being coerced into doing so. | Yes | No |
| I am aware that my answers and opinions will remain confidential. | Yes | No |
| I understand that I can withdraw from the study at any time without any consequences. | Yes | No |
| I need data costs to be covered in order to participate in the study | Yes | No |
| I give consent to be contacted to participate in an interview that will explore MPH career paths, competencies and recommendations | Yes | No |
| By completing this questionnaire I confirm that I am willing to participate in this study. | Yes | No |
| I understand that only the items indicated in the questionnaire will be included in the data base | Yes | No |

3.	COMPETENCIES

We now would like you to assess whether you feel you have the following competencies, whether they are applicable to your work, and if you got them from the MPH. They are divided into the core disciplines taught in many MPH programs.

Please select a number on the scale that you think applies to you.

**DOMAIN 1. PUBLIC HEALTH SCIENCE SKILLS**

| **DETAILED COMPETENCIES** | **Level of confidence (in possessing this competency)**  **1 Not confident**  **2 A little confident**  **3 Moderately confident**  **4 Very confident** | **Extent to which MPH enabled acquisition of competency**   1. **Not due to MPH** 2. **MPH enabled a little** 3. **MPH enabled moderately** 4. **MPH enabled substantially** | **Applicability of Competence to current or most recent job**   1. **Not applicable** 2. **Slightly applicable** 3. **Moderately applicable** 4. **Very applicable** |
| --- | --- | --- | --- |
| 1. I can apply the public health sciences of biostatistics, epidemiology, to public health policies and programs |  |  |  |
| 2. I can apply basic environmental health skills to public health policies and programs. |  |  |  |
| 3. I can apply health service management skills to public health programs. |  |  |  |
| 4. I can apply the public health approach in my local context, health system and other social sectors. |  |  |  |
| 5. I can analyse the health status of populations and identify population health problems, risk factors for ill health and their related social determinants. |  |  |  |

**DOMAIN 2.	HEALTH PROMOTION COMPETENCIES**

| **DETAILED COMPETENCIES** | **Level of *confidence* (in possessing this competency)**  **1 Not confident**  **2 A little confident**  **3 Moderately confident**  **4 Very confident** | **Extent to which *MPH* *enabled* *acquisition* of competency**   1 **Not due to MPH** 2 **MPH enabled a little** 3 **MPH enabled moderately** 4 **MPH enabled substantially** | ***Applicability* of Competence to current or most recent job**   1. **Not applicable** 2. **Slightly applicable** 3. **Moderately applicable** 4. **Very applicable** |
| --- | --- | --- | --- |
| 1. I can outline the role of the media in health promotion. |  |  |  |
| 2. I can apply the levels of disease prevention approach(primary secondary, tertiary) to a range of health conditions. |  |  |  |
| 3. I am familiar with major health promotion policies and strategies in at least one African country. |  |  |  |
| 4. I can plan health promotion activities based on research. |  |  |  |
| 5. When needed, I can generate factual information to counteract industry marketing in relation to nutrition, tobacco cessation, reducing alcohol consumption in at least one African country. |  |  |  |
| 6. I can design educational materials using local resources in at least one African country to communicate and promote health. |  |  |  |
| 7. I can engage the media in planning health promotion activities |  |  |  |

**DOMAIN 3	MONITORING AND EVALUATION (M&E)**

| **DETAILED COMPETENCIES** | **Level of confidence (in possessing this competency)**  **1 Not confident**  **2 A little confident**  **3 Moderately confident**  **4 Very confident** | **Extent to which MPH enabled acquisition of competency**   1 **Not due to MPH** 2 **MPH enabled a little** 3 **MPH enabled moderately** 4 **MPH enabled substantially** | **Applicability of Competence to current or most recent job**   1. **Not applicable** 2. **Slightly applicable** 3. **Moderately applicable** 4. **Very applicable** |
| --- | --- | --- | --- |
| 1. I can make use of M & E data to support decision making, advocacy and other purposes related to Public Health. |  |  |  |
| 2. I can design appropriate indicators to monitor and evaluate public health programs and interventions. |  |  |  |
| 3. I can *monitor* public health policies, interventions and regulations using standard monitoring and evaluation indicators of input, process and outcome. |  |  |  |
| 4. I can *evaluate* public health policies, interventions and regulations using standard monitoring and evaluation indicators of input, process, outcome and impact. |  |  |  |
| 5. I can evaluate public health policies, interventions and regulations using additional techniques such as stakeholder interviews. |  |  |  |
| 6. I can perform community diagnoses to identify the common health challenges faced by a population |  |  |  |
| 7. I can develop a practical plan to address locally identified health challenges. |  |  |  |

**DOMAIN 4	HEALTH POLICY COMPETENCIES**

| **DETAILED COMPETENCIES** | **Level of confidence (in possessing this competency)**  **1 Not confident**  **2 A little confident**  **3 Moderately confident**  **4 Very confident** | **Extent to which MPH enabled acquisition of competency**   1. **Not due to MPH** 2. **MPH enabled a little** 3. **MPH enabled moderately** 4. **MPH enabled substantially** | **Applicability of Competence to current or most recent job**   1. **Not applicable** 2. **Slightly applicable** 3. **Moderately applicable** 4. **Very applicable** |
| --- | --- | --- | --- |
| 1. I can evaluate policy options to address identified health problems. |  |  |  |
| 2. I can assess the feasibility of public health policies/programs in diverse community contexts, using the appraisal of evidence. |  |  |  |
| 3. I can participate in developing context sensitive health policies |  |  |  |
| 4. I can participate in developing context sensitive strategic plans. |  |  |  |
| 5. I can implement public health policies. |  |  |  |

**DOMAIN 5	COMMUNICATION COMPETENCIES**

| **DETAILED COMPETENCIES** | **Level of confidence (in possessing this competency)**  **1 Not confident**  **2 A little confident**  **3 Moderately confident**  **4 Very confident** | **Extent to which MPH enabled acquisition of competency**   1. **Not due to MPH** 2. **MPH enabled a little** 3. **MPH enabled moderately** 4. **MPH enabled substantially** | **Applicability of Competence to current or most recent job**   1. **Not applicable** 2. **Slightly applicable** 3. **Moderately applicable** 4. **Very applicable** |
| --- | --- | --- | --- |
| 1. I can communicate concisely in *writing* for different audiences. |  |  |  |
| 2. I can effectively communicate *orally* for a range of different audiences. |  |  |  |
| 3. I can facilitate and integrate contributions to health policy and programs from a wide range of stakeholders |  |  |  |
| 4. I can listen and respond in a way that fits the audience. |  |  |  |
| 5. I am able to convey health data and information to a professional audience using appropriate methods/means. |  |  |  |
| 6. I can convey health data and information to the general public using appropriate methods/means. |  |  |  |
| 7. I can contribute to advocacy of new and existing health policies. |  |  |  |

**DOMAIN 6	CONTEXT SPECIFIC COMPETENCIES**

| **DETAILED COMPETENCIES** | **Level of confidence (in possessing this competency)**  **1 Not confident**  **2 A little confident**  **3 Moderately confident**  **4 Very confident** | **Extent to which MPH enabled acquisition of competency**   1. **Not due to MPH** 2. **MPH enabled a little** 3. **MPH enabled moderately** 4. **MPH enabled substantially** | **Applicability of Competence to current or most recent job**   1. **Not applicable** 2. **Slightly applicable** 3. **Moderately applicable** 4. **Very applicable** |
| --- | --- | --- | --- |
| 1. I know how gender, cultural, social, economic, political and behavioral factors affect the accessibility, availability, acceptability and delivery of public health services and programs. |  |  |  |
| 2. I know how history, power, privilege and structural inequality have produced health disparities.. |  |  |  |
| 3. I incorporate a social determinants of health approach to assessing health needs and interventions. |  |  |  |
| 4. I know how health system values and organization affect the quality of health care delivery, which in turn influences the quality of people's lives. |  |  |  |

**DOMAIN 7	COMMUNITY AND INTERSECTORAL COMPETENCIES**

| **DETAILED COMPETENCIES** | **Level of confidence (in possessing this competency)**  **1 Not confident**  **2 A little confident**  **3 Moderately confident**  **4 Very confident** | **Extent to which MPH enabled acquisition of competency**   1. **Not due to MPH** 2. **MPH enabled a little** 3. **MPH enabled moderately** 4. **MPH enabled substantially** | **Applicability of Competence to current or most recent job**   1. **Not applicable** 2. **Slightly applicable** 3. **Moderately applicable** 4. **Very applicable** |
| --- | --- | --- | --- |
| 1. I can engage community actors to promote health. |  |  |  |
| 2. I am able to partner with diverse stakeholders. |  |  |  |

**DOMAIN 8	PLANNING AND MANAGEMENT COMPETENCIES**

| **DETAILED COMPETENCIES** | **Level of confidence (in possessing this competency)**  **1 Not confident**  **2 A little confident**  **3 Moderately confident**  **4 Very confident** | **Extent to which MPH enabled acquisition of competency**   1. **Not due to MPH** 2. **MPH enabled a little** 3. **MPH enabled moderately** 4. **MPH enabled substantially** | **Applicability of Competence to current or most recent job**   1. **Not applicable** 2. **Slightly applicable** 3. **Moderately applicable** 4. **Very applicable** |
| --- | --- | --- | --- |
| 1. I can use evidence to plan interventions that address local priority health issues. |  |  |  |
| 2. I can identify whether the principles of the primary health care model (as described in Alma Ata Declaration) are being adhered to in planning and managing public health programs or interventions. |  |  |  |

| 3. I can plan and implement health interventions and services using the domains of input, process, outcome and impact. |  |  |  |
| --- | --- | --- | --- |
| 4. I contribute to managing and evaluating health information systems that includes human, financial and logistic resources. |  |  |  |
| 5. I can utilize cost-effectiveness, cost-benefit, and cost-utility analyses in programmatic prioritization and decision making. |  |  |  |

**DOMAIN 9	CRISIS MANAGEMENT AND EMERGENCY PREAPREDNESS**

| **DETAILED COMPETENCIES** | **Level of confidence (in possessing this competency)**  **1 Not confident**  **2 A little confident**  **3 Moderately confident**  **4 Very confident** | **Extent to which MPH enabled acquisition of competency**   1. **Not due to MPH** 2. **MPH enabled a little** 3. **MPH enabled moderately** 4. **MPH enabled substantially** | **Applicability of Competence to current or most recent job**   1. **Not applicable** 2. **Slightly applicable** 3. **Moderately applicable** 4. **Very applicable** |
| --- | --- | --- | --- |
| 1. I can describe the processes involved in identifying as rapidly as possible the (possibly novel) agents responsible for a disease outbreak and their epidemiological characteristics. |  |  |  |
| 2. I can conduct outbreak investigations to identify pathogens/other agents, characterize affected population, and sources of exposure. |  |  |  |
| 3. I can use event-based and indicator-based surveillance systems to detect health threats. |  |  |  |
| 4. I can critically appraise international public health alerts to assess the implications for my local context. |  |  |  |

**DOMAIN 10	ACADEMIC/RESEARCH COMPETENCIES**

| **DETAILED COMPETENCIES** | **Level of confidence (in possessing this competency)**  **1 Not confident**  **2 A little confident**  **3 Moderately confident**  **4 Very confident** | **Extent to which MPH enabled acquisition of competency**   1. **Not due to MPH** 2. **MPH enabled a little** 3. **MPH enabled moderately** 4. **MPH enabled substantially** | **Applicability of Competence to current or most recent job**   1. **Not applicable** 2. **Slightly applicable** 3. **Moderately applicable** 4. **Very applicable** |
| --- | --- | --- | --- |
| 1. I can review available literature to inform health decisions. |  |  |  |
| 2. I can interpret findings from research and communicate these findings in simple terms to the public. |  |  |  |
| 3. I can develop a sound research proposal with a methodology that upholds research ethics standards. |  |  |  |
| 4. I can identify conflict of interest in public health research. |  |  |  |
| 5. I can appropriately reference literature sources of public health data and information. |  |  |  |
| 6. I can put together a research grant application to source funding to support research. |  |  |  |
| 7. I can develop a research budget. |  |  |  |

**DOMAIN 11	LEADERSHIP COMPETENCIES**

| **DETAILED COMPETENCIES** | **Level of confidence (in possessing this competency)**  **1 Not confident**  **2 A little confident**  **3 Moderately confident**  **4 Very confident** | **Extent to which MPH enabled acquisition of competency**   1. **Not due to MPH** 2. **MPH enabled a little** 3. **MPH enabled moderately** 4. **MPH enabled substantially** | **Applicability of Competence to current or most recent job**   1. **Not applicable** 2. **Slightly applicable** 3. **Moderately applicable** 4. **Very applicable** |
| --- | --- | --- | --- |
| 1. I can adapt my work environment or setting to cope with changes caused by public health emergencies such as the COVID-19 pandemic |  |  |  |
| 2. I can apply team building skills in my workplace |  |  |  |
| 3. I can apply conflict resolution skills in my workplace |  |  |  |
| 4. I can describe the attributes of a good public health leader |  |  |  |
| 5. I can apply human rights principles and equity indecision making |  |  |  |

**Scoring system for questionnaire items***

| **Competency domain** | **Items** | **Confidence (summation of scores)** | **Acquisition through MPH? (summation of scores)** | **Applicable to work  (summation of scores)** |
| --- | --- | --- | --- | --- |
| **Scale** |  | 0. Not confident 1. A little confident 2. Moderately confident 3. Very confident | 0. Not due to MPH 1. MPH enabled a little 2. MPH enabled moderately 3. MPH enabled substantially | 0. Not applicable 1. Slightly applicable 2. Moderately applicable 3. Very applicable |
| 1. **Public Health Science skills** | 5 | 0-15 | 0-15 | 0-15 |
| 2. **Health Promotion competencies** | 7 | 0-21 | 0-21 | 0-21 |
| 3. **Monitoring & Evaluation** | 7 | 0-21 | 0-21 | 0-21 |
| 4. **Health Policy** | 5 | 0-15 | 0-15 | 0-15 |
| 5. **Communication for a range of audiences** | 7 | 0-15 | 0-15 | 0-15 |
| 6. **Context sensitive** | 4 | 0-12 | 0-12 | 0-12 |
| 7. **Community & intersectoral** | 2 | 0-6 | 0-6 | 0-6 |
| 8. **Planning & management** | 5 | 0-15 | 0-15 | 0-15 |
| 9. **Crisis Mx & Emergency preparedness** | 4 | 0-12 | 0-12 | 0-12 |
| 10. **Academic/research** | 7 | 0-15 | 0-15 | 0-15 |
| 11. **Leadership** | 5 | 0-15 | 0-15 | 0-15 |

* Each item scored between 1 and 4. This was **recoded**: ‘not’=0 and very or substantially=3. A percentage score for each median and Interquartile range was calculated by dividing the median by the total possible.

4. IMPROVING THE MPH PROGRAM

We would like to find out your reflections on how the MPH program can be improved. Please rate your level of agreement with the following statements.

The MPH program can be improved by:

|  | **Roles** | **1 Strongly disagree** | **2 Disagree** | **3 Neither disagree nor agree** | **4 Agree** | **5 Strongly agree** |
| --- | --- | --- | --- | --- | --- | --- |
| 1 | Offering practical or field training to students). |  |  |  |  |  |
| 2 | Placing emphasis on the development of problem-solving skills (E.g., asking students to come up with a plan to deal with pandemics like COVID-19). |  |  |  |  |  |
| 3 | Having students mentored by faculty members or senior students. |  |  |  |  |  |
| 4 | Any other reflections on how the MPH program can be improved? |  |  |  |  |  |
