# Supplementary material for: Self-perceived competencies of Master of Public Health graduates and recommendations for programs in South Africa
Source: PLoS One. 2026 Oct 16;21(10):e0360789. doi: 10.1371/journal.pone.0360789 (PMC13674699; doi:10.1371/journal.pone.0360789)
Supplement: Supplementary file 2 [file pone.0360789.s002.docx]

**Supplementary figures and tables**

**1.	Table of Shapiro-Wilks p-values for all domains by sphere**

|  | **Spheres** | | |
| --- | --- | --- | --- |
| **Competency domain** | **Applicable to work** | **Acquired from MPH** | **Confidence in use** |
| **Public Health Sciences** | 0.0003 | 0.0384 | 0.0006 |
| **Health promotion** | 0.0140 | 0.0463 | 0.0018 |
| **Monitoring & Evaluation** | 0.0000 | 0.0005 | 0.0001 |
| **Health Policy** | 0.0010 | 0.0388 | 0.0004 |
| **Communication** | 0.0000 | 0.0026 | 0.0000 |
| **Context sensitive** | 0.0000 | 0.0001 | 0.0000 |
| **Community & intersectoral** | 0.0000 | **0.5248** | 0.0000 |
| **Planning &Management** | 0.0000 | 0.0400 | 0.0000 |
| **Crisis & Emergency** | 0.0050 | 0.0007 | **0.7560** |
| **Academic** | 0.0001 | 0.0001 | 0.0000 |
| **Leadership** | 0.0000 | 0.0000 | 0.0000 |

All but two (p-values in bold) are significant., meaning the variables are not normally distributed.

**2.	Chronbach alphas calculated for self perceived competency domains by sphere: applicability to work, derived through MPH and confidence**

|  | **Competency domain** | **Items** | **Applicable to work** | **Through MPH?** | **Confidence in use** |
| --- | --- | --- | --- | --- | --- |
| 1 | **Public Health Science skills** | 5 | 0,77 | 0,81 | 0,72 |
| 2 | **Health Promotion competencies** | 7 | 0,92 | 0,89 | 0,91 |
| 3 | **Monitoring & Evaluation** | 7 | 0,93 | 0,94 | 0,93 |
| 4 | **Health Policy** | 5 | 0,94 | 0,91 | 0,91 |
| 5 | **Communication** | 7 | 0,95 | 0,90 | 0,91 |
| 6 | **Context sensitive** | 4 | 0,92 | 0,90 | 0,89 |
| 7 | **Community & intersectoral** | 2 | 0,91 | 0,50 | 0,65 |
| 8 | **Planning & management** | 5 | 0,92 | 0,92 | 0,89 |
| 9 | **Crisis Mx & Emergency preparedness** | 4 | 0,95 | 0,92 | 0,94 |
| 10 | **Academic/research** | 7 | 0,85 | 0,81 | 0,94 |
| 11 | **Leadership** | 5 | 0,94 | 0,88 | 0,86 |

**3**.	**Recruitment and response rates**

a.	Recruitment of potential respondents by institution (N=710; n=237)

Number

| University | Graduates 2012-2016 | with emails/phone numbers | contact attempted | responded (as at 22 Jan 2023) |
| --- | --- | --- | --- | --- |
| 1 | 64 | 64 | 64 | 45 |
| 2 | 66 | 66 | 66 | 25 |
| 3 | 153 | 153 | 153 | 63 |
| 4 | 47 | 47 | 38 | 11 |
| 5 | 138 | 138 | 138 | 25 |
| 6 | 96 | 94 | 94 | 39 |
| 7 | 146 | 127 | 105 | 29 |

b. Response rate by institution*

| University | Percentage |
| --- | --- |
| 1 | 70.3125 |
| 2 | 37.8788 |
| 3 | 41.1765 |
| 4 | 28.9474 |
| 5 | 18.1159 |
| 6 | 41.4894 |
| 7 | 19.863 |

* Respondents/contact attempted

**4.	Respondents scoring of own competencies *not* relevant to work, *not* obtained from MPH and *not* confident by item***

|  | **Items** | **n (mean)** | **Item 1 %**  **(CI)** | **Item 2 %** | **Item 3 %** | **Item 4 %** | **Item 5 %** | **Item 6 %** | **Item 7 %** | **Median %** |
| --- | --- | --- | --- | --- | --- | --- | --- | --- | --- | --- |
|  |  |  |  | **(CI)** | **(CI)** | **(CI)** | **(CI)** | **(CI)** | **(CI)** | **(IQR)** |
| **Not applicable to work** | | | | | | | | | | |
| Public Health science skills | 5 | 166 | 7.2 | 18.3 | 7.9 | 2.4 | 4.2 |  | | 7.2 |
|  |  |  | (4.1-12.3) | (1.3-25.0) | (4.6-13.4) | (0.9-6.3) | (2.0-8.6) |  |  | (4.2-7.9) |
| Health Promotion competencies | 7 | 154 | 8.3 | 2.6 | 5.1 | 4.6 | 8.6 | 4.5 | 11.8 | 5.2 |
|  |  |  | (4.8-13.8) | (1.0-6.7) | (2.6-10.0) | (2.2-9.4) | (5.0-14.3) | (2.2-9.2) | (7.6-18.1) | (4.5-8.6) |
| Monitoring & Evaluation | 7 | 151 | 4.0 | 4.6 | 4.8 | 4.0 | 6.7 | 7.9 | 6.0 | 4.7 |
|  |  |  | (1.8-8.6) | (2.2-9.5) | (2.3-9.7) | (1.8-8.7) | (3.6-12.1) | (4.5-13.6) | (3.1-11.1) | (4.0-6.7) |
| Health Policy | 5 | 137 | 7.4 | 10.3 | 10.2 | 8.8 | 12.4 |  | | 10.2 |
|  |  |  | (4.0-13.2) | (6.2-16.7) | (6.1-16.6) | (5.0-14.9) | (7.8-19.2) |  |  | (8.8-10.3) |
| Communication | 7 | 137 | 1.3 | 1.5 | 3.7 | 2.9 | 2.2 | 2.9 | 6.5 | 2.9 |
|  |  |  | (8.3-19.9) | (0.4-5.7) | (1.5-8.7) | (1.1-7.6) | (0.7-6.8) | (1.1-7.6) | (3.4-12.1) | (1.5-3.7) |
| Context sensitive | 4 | 139 | 4.3 | 4.3 | 3.6 | 5 |  | | | 4.3 |
|  |  |  | (1.9-9.4) | (1.9-9.2) | (1.5-8.5) | (2.4-10.3) |  |  |  | (4.1-6.4) |
| Community & intersectional | 2 | 124 | 6.4 | 4.1 |  | | | | | 5.2 |
|  |  |  | (3.2-12.4) | (1.7-9.5) |  |  |  |  |  | (4.1-6.4) |
| Planning & management (Mx) | 5 | 125 | 7 | 10.2 | 10.3 | 11 | 15.7 |  | | 10.3 |
|  |  |  | (3.7-13.1) | (5.9-16.8) | (6.0-17.1) | (6.6-17.9) | (10.3-23.3) |  |  | (10.2-11.0) |
| Crisis Mx & Emerg preparedness | 4 | 125 | 15.1 | 23.8 | 25.6 | 16.0 |  | | | 19.9 |
|  |  |  | (9.8-22.6) | (17.1-32.1) | (18.6-34.1) | (10.5-23.6) |  |  |  | (15.5-24.7) |
| Academic/research | 7 | 126 | 0 | 0 | 0 | 0 | 0 | 4.8 | 4.0 | 0.0 |
|  |  |  |  |  |  |  |  | (2.1-10.3) | (1.6-9.3) | (0.0-4.0) |
| Leadership | 5 | 124 | 2.4 | 0.8 | 2.4 | 5.6 | 1.6 |  | | 2.4 |
|  |  |  | (0.8-7.3) | (0.1-5.6) | (0.8-7.3) | (2.7-11.4) | (0.4-6.3) |  |  | (1.6-2.4) |

| **Not obtained from MPH** | | | | | | | | | | |
| --- | --- | --- | --- | --- | --- | --- | --- | --- | --- | --- |
| Public Health science skills | 5 | 166 | 3.0 | 16.6 | 6.8 | 0.0 | 1.8 |  |  | 6.7 |
|  |  |  | (1.2-7.0) | (11.6-23.2) | (3.8-11.8) |  | (0.6-5.4) |  |  | (3.0-8.4) |
| Health Promotion competencies | 7 | 151 | 18.2 | 10.6 | 9.9 | 11.3 | 10.6 | 11.6 | 17.1 | 11.3 |
|  |  |  | (12.8-25.2) | (6.6-16.7) | (6.0-15.9) | (7.1-17.4) | (6.6-16.7) | (7.4-17.8) | (11.9-24.0) | (10.6-17.1) |
| Monitoring & Evaluation | 7 | 150 | 12.7 | 14.7 | 14.1 | 12.8 | 14.1 | 18.0 (12.6-25.1) | 12.0 | 14.1 |
|  |  |  | (7.2-19.1) | (9.8-21.4) | (9.3-20.7) | (8.2-19.2) | (9.3-20.7) |  | (7.7-18.3) | (12.7-14.7) |
| Health Policy | 5 | 135 | 5.2 | 6.7 | 7.4 | 8.8 | 7.4 |  | | 7.4 |
|  |  |  | (2.5-10.6) | (3.5-12.4) | (4.0-13.3) | (5.0-15.0) | (4.0-13.3) |  |  | (6.7-7.4) |
| Communication | 7 | 137 | 13.0 | 8.8 | 13.3 | 12.5 | 9.0 | 7.3 | 10.2 | 11.0 |
|  |  |  | (8.3-19.9) | (5.0-14.9) | (8.5-20.3) | (7.9-19.3) | (5.2-15.3) | (3.9-13.1) | (6.1-16.6) | (9.8-13.0) |
| Context sensitive | 4 | 138 | 5.1 | 8.0 | 5.1 | 6.5 |  | | | 5.8 |
|  |  |  | (2.4-10.3) | (4.4-13.9) | (2.4-10.4) | (3.4-12.1) |  |  |  | (5.1-7.3) |
| Community & intersectional | 2 | 123 | 12.3 | 9.8 |  | | | | | 11.0 |
|  |  |  | (7.5-19.5) | (5.6-16.5) |  |  |  |  |  | (9.8-12.3) |
| Planning & management (Mx) | 5 | 125 | 8.7 | 12.7 | 12.8 | 16.8 | 24.8 |  | | 12.8 |
|  |  |  | (4.9-15.2) | (7.9-19.8) | (7.9-20.0) | (11.2-24.5) | (17.9-33.2) |  |  | (12.7-16.8) |
| Crisis Mx & Emerg preparedness | 4 | 125 | 29.4 | 34.1 | 32.0 | 30.7 |  | | | 31.3 |
|  |  |  | (22.0-38.0) | (26.3-42.9) | (24.3-40.8) | (23.1-39.4) |  |  |  | (30.0-33.1) |
| Academic/research | 7 | 126 | 0.8 | 3.2 | 0.8 | 4.0 | 0.8 | 15.9 | 20.6 | 3.2 |
|  |  |  | (0.1-5.6) | (1.2-8.3) | (0.1-5.6) | (1.6-9.3) | (0.1-5.6) | (10.4-23.4) | (14.4-28.7) | (0.8-15.9) |
| Leadership | 5 | 125 | 26.6 | 28.8 | 32.3 | 20.0 | 21.0 |  | | 26.6 |
|  |  |  | (19.5-35.2) | (21.4-37.5) | (24.5-41.1) | (13.8-28.1) | (14.6-29.1) |  |  | (21.0-28.8) |

| **Not confident possessed** | | | | | | | | | | |
| --- | --- | --- | --- | --- | --- | --- | --- | --- | --- | --- |
| Public Health Science skills | 5 | 166 | 3.6 | 13.9 | 4.9 | 1.2 | 1.8 |  |  | 3.6 |
|  |  |  | (1.6-7.8) | (9.3-20.1) | (2.4-9.5) | (0.3-4.8) | (0.6-5.4) |  |  | (1.8-4.9) |
| Health Prom competencies | 7 | 152 | 13.8 | 7.8 | 7.8 | 12.5 | 16 | 13.1 | 18.4 | 13.1 |
|  |  |  | (9.1-20.3) | (4.5-13.4) | (4.5-13.4) | (8.1-18.9) | (10.9-22.9) | (8.5-19.5) | (13.0-25.5) | (7.8-16.0) |
| Monitor & Evaluation | 7 | 149 | 3.4 | 6.7 | 5.4 | 4.1 | 8 | 6.7 | 3.3 | 5.4 |
|  |  |  | (1.4-7.9) | (3.6-12.1) | (2.7-10.4) | (1.8-8.8) | (4.6-13.6) | (3.6-12.0) | (1.4-7.8) | (3.4-6.1) |
| Health Policy | 5 | 136 | 4.4 | 5.1 | 5.8 | 5.9 | 5.2 |  | | 5.1 |
|  |  |  | (2.0-9.6) | (2.4-10.4) | (2.9-11.3) | (2.9-11.4) | (2.5-10.5) |  |  | (5.1-5.9) |
| Communication | 7 | 137 | 0.7 | 1.5 | 2.9 | 1.5 | 1.5 | 0.7 | 3.7 | 1.5 |
|  |  |  | (0.1-5.1) | (3.6-5.8) | (1.1-7.7) | (0.3-5.8) | (0.4-5.9) | (0.1-5.1) | (1.5-8.6) | (0.7-2.9) |
| Context sensitive | 4 | 137 | 2.2 | 2.2 | 1.5 | 2.9 |  | | | 2.2 |
|  |  |  | (0.7-6.6) | (0.7-6.7) | (0.4-5.8) | (1.1-7.6) |  |  |  | (1.8-2.5) |
| Community & intersectional | 2 | 124 | 4.1 | 2.4 |  | | | | | 3.2 |
|  |  |  | (1.7-9.5) | (0.8-7.3) |  |  |  |  |  | (2.4-4.1) |
| Planning & management (Mx) | 5 | 125 | 1.6 | 2.4 | 4 | 3.2 | 9.6 |  | | 3.2 |
|  |  |  | (0.4-6.2) | (0.8-7.2) | (1.7-9.4) | (1.2-8.3) | (5.5-16.3) |  |  | (2.4-4.0) |
| Crisis Mx & Emerg prepared | 4 | 127 | 16.5 | 22.7 | 22.1 | 15.8 |  | | | 19.3 |
|  |  |  | (11.0-24.2) | (16.1-30.8) | (15.6-30.2) | (10.3-23.3) |  |  |  | (16.1-22.4) |
| Academic /research | 7 | 128 | 1.6 | 0 | 3.9 | 6.3 | 2.4 | 6.3 | 10.2 | 3.9 |
|  |  |  | (0.4-6.1) |  | (1.6-9.2) | (3.1-12.1) | (0.8-7.2) | (3.1-12.1) | (5.9-16.8) | (1.6-6.3) |
| Leadership | 5 | 126 | 2.4 | 4.7 | 3.2 | 7.1 | 4.8 |  | | 4.7 |
|  |  |  | (0.8-7.3) | (2.1-10.2) | (1.2-8.3) | (3.7-13.2) | (2.1-10.4) |  |  | (3.2-4.8) |
